# Revision of the genus *Philonome* Chambers and its proposed reassignment to the family Tineidae (Lepidoptera, Tineoidea)

**DOI:** 10.3897/zookeys.494.8748

**Published:** 2015-04-06

**Authors:** Jae-Cheon Sohn, Donald R. Davis, Carlos Lopez-Vaamonde

**Affiliations:** 1 Department of Entomology, Smithsonian Institution, National Museum of Natural History, 10th & Constitution NW, Washington, DC 20560, USA; 2 Department of Entomology, 4112 Plant Sciences Building, University of Maryland, College Park, MD 20742, USA; 3 INRA, UR0633 Zoologie Forestière, F-45000, Orléans, France; 4 Institut de Recherche sur la Biologie de l’Insecte (IRBI), CNRS UMR 7261, Université François Rabelais, Faculté des Sciences et Techniques, 37200 Tours, France

**Keywords:** *Argyresthia*, Argyresthiidae, Bucculatricidae, COI, DNA barcoding, *Eurynome*, Lyonetiidae, new species, New World

## Abstract

The New World genus *Philonome* Chambers, 1874 is revised. This genus comprises twelve species, seven of which are described as new: two species, *Philonome nigrescens* **sp. n.** and *Philonome wielgusi* **sp. n.**, from the United States; four species, *Philonome albivittata* **sp. n.**, *Philonome curvilineata* **sp. n.**, *Philonome kawakitai* **sp. n.**, and *Philonome lambdagrapha* **sp. n.**, from French Guiana; and one species, *Philonome penerivifera* **sp. n.**, from Brazil. Lectotypes are designated for *Philonome clemensella* Chambers, 1874 and *Philonome rivifera* Meyrick, 1915. Partially on evidence of their head morphology and particularly from molecular evidence, the genus *Philonome*, previously associated with Bucculatricidae or Lyonetiidae, is reassigned to Tineidae. A possible systematic position of *Philonome* within Tineidae is discussed. *Eurynome* Chambers, 1875, is synonymized with *Argyresthia* Hübner, 1825 (Argyresthiidae). Photographs of adults and illustrations of genitalia, when available, are provided for all described species of *Philonome* and two species previously misplaced in *Philonome*, *Argyresthia luteella* (Chambers, 1875) and *Elachista albella* (Chambers, 1877). In addition, DNA barcodes were used for the delimitation of most species.

## Introduction

The monobasic genus *Philonome* was proposed by Chambers in 1874 for *Philonome clemensella* Chambers. Chambers (1875) later proposed a supposedly allied genus, *Eurynome*, also based on a single species, *Eurynome luteella* Chambers, and in 1877, added another congener, *Eurynome albella* Chambers. *Eurynome*, however, was recognized as a homonym and later replaced by *Busckia* Dyar, 1903.

**Figure 1.**
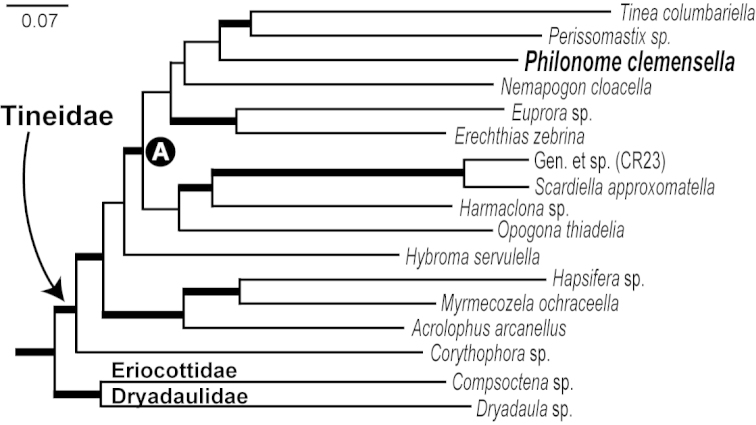
Maximum likelihood phylogeny of Tineidae *s. l.* extracted from Sohn et al. (2013), based on 27 nuclear genes. Branches in bold indicate the > 70% bootstrapping support from at least one analysis attempted by Sohn et al. (2013). The ‘A’ in closed circle represents a well-supported subclade of Tineidae in which *Philonome clemensella* is included.

Chambers (1875, 1877, 1880) assigned *Philonome* to the Tineina, a conventional group name to accommodate primitive Microlepidoptera, and he further suggested that the genus is allied to *Bucculatrix* Zeller, 1839. The putative association between *Philonome* and *Bucculatrix* has been repeatedly expressed by subsequent researchers such as Meyrick (1915, 1920) and Forbes (1923). Barnes and McDunnough (1917) included *Philonome* under Lyonetiidae, together with *Bucculatrix*, followed by Forbes (1923), but they treated *Busckia* (= *Eurynome* Chambers) as a genus of Elachistidae. McDunnough (1939) transferred *Busckia* to Lyonetiidae and synonymized it with *Philonome*. Sohn et al. (2013) conducted a molecular phylogeny of Yponomeutoidea (to which Lyonetiidae belongs), including *Philonome clemensella*, and found that the species is nested within the Tineidae (Fig. 1). However, the tineid association of *Philonome* has been so far supported only by molecular data, not yet by morphological evidence.

*Philonome* currently includes six species, which occur exclusively in the New World: two from the Nearctic Region and four from the Neotropical Region. *Eurynome albella* Chambers (Figs 17, 70), known only from the unique holotype collected at Edgerton (38°57'24"N, 104°50'6"W; at ~ 6500 feet elevation), El Paso Co., Colorado, was once treated as *Philonome* (McDunnough 1939), but it was later assigned to *Elachista* of Elachistidae (Kaila 1999). Kaila (1999) found that the name *Elachista albella* (Chambers) had been preoccupied and hence he proposed a replacement name, *Elachista dasycara*. Chambers (1874, 1877) characterized *Philonome* and *Eurynome* on superficial appearance and wing venation. The adults resemble some species of *Bucculatrix* in wing pattern, notably *Bucculatrix adelpha* Braun, 1963, or *Bucculatrix angustata* Frey & Boll, 1876. However, *Philonome* differs from *Bucculatrix* in having an elongate, telescopic ovipositor and lacking an androconial scale pocket on the male abdomen (Braun 1963; Kobayashi et al. 2010). This suggests that their resemblance is due to convergence. The biology of *Philonome* is essentially unknown. Forbes (1923) stated that *Philonome clemensella* have been collected from hickory and linden trees. His statement, however, was based on the ambiguous label data of specimens from the United States National Museum of Natural History. No additional observation of the larvae of *Philonome clemensella* has been reported from these trees.

The goals of this paper are to redefine the generic characteristics of *Philonome*, to describe seven new species from the Nearctic and Neotropical Regions, to transfer a misplaced species, “Philonome” luteella to its correct genus, *Argyresthia*, and to provide morphological evidence of the tineid relationship of *Philonome*, which has been suggested from a recent molecular phylogenetic study (Sohn et al. 2013).

## Materials and methods

Pinned specimens from five institutional collections were examined. The abbreviations of these depositories are as follows:

BMNH Natural History Museum (formerly British Museum of Natural History), London, UK;

MCZ Museum of Comparative Zoology, Harvard University, Cambridge, USA;

MSU Mississippi Entomological Museum, Mississippi State University, Starkville, Mississippi, USA;

USNM National Museum of Natural History (formerly United States Museum of Natural History), Washington DC, USA;

VOB Vitor O. Becker, Instituto Uiraçu, Camacan, Brazil.

Other abbreviations include:

ex. example, the specimens whose sex cannot be determined;

Co. county;

GSN genitalia slide number;

WSN wing slide number.

Selected specimens were dissected for genitalia and abdominal structures, following Clarke (1941), except that Chlorazol black was used for staining. Dissected genitalia were mounted on microscope slides in Euparal resin (BioQuip Products Inc.) or Canada balsam. Pinned specimens were examined under a Leica MZ APO stereoscope. Slide-mounted specimens were examined under a Leica LEITZ-DMRX microscope. All illustrations were drawn from dissections temporarily stored in glycerin, which were later permanently embedded in mounting medium. Terms for genitalia and wing venation follow Klots (1970) and Wootton (1979), respectively. The 8^th^ abdominal segment is abbreviated as A8 in the descriptions. Verbatim label data are given for primary types. Additional data by the present authors are given in brackets.

DNA was extracted from hind legs of dried specimens. DNA barcodes (658 bp of the COI mitochondrial gene) were generated at the Canadian Centre for DNA Barcoding (CCDB, Guelph). A total of seven specimens were sequenced (Table 1), all collected in French Guiana by the third author (CLV). These newly generated barcodes were compared to five DNA-barcodes of *Philonome clemensella* (Table 1), one (jflandry0875) available at the Barcode of Life Data Systems (BOLD; www.boldsystems.org; also see Ratnasingham and Hebert 2007) and the other four unpublished. Barcode data were analysed using the analytical tools of BOLD such as Neighbour Joining and pairwise genetic distance matrix.

**Table 1.** Specimens used for the DNA barcoding analysis. Both the Process ID and sample ID codes are unique identifiers linking the record in the BOLD database and the voucher specimen from which the sequence is derived. Additional collecting and specimen data are accessible in BOLD’s data set (http://dx.doi.org/10.5883/DS-PHILONO) as well as GenBank (http://www.ncbi.nlm.nih.gov/genbank/).

Species	Process ID	Sample ID	Country	Accession number (NCBI, GENBANK)
*Philonome clemensella*	MEC875-04	jflandry0875	Canada	GU096008
*Philonome clemensella*	MNAL543-10	CNCLEP00035968	Canada	KP696787
*Philonome clemensella*	MNAI712-09	CNCLEP00038457	Canada	GU692620
*Philonome clemensella*	MNAI218-09	CNCLEP00042501	USA	GU693088
*Philonome clemensella*	MNAI241-09	CNCLEP00042524	USA	GU693064
*Philonome curvilineata*	LNOUA586-10	CLV68110	French Guiana	HQ571412
*Philonome euryarga*	LNOUA669-10	CLV76410	French Guiana	HQ571490
*Philonome albivittata*	LNOUA849-10	CLV94410	French Guiana	HQ571657
*Philonome albivittata*	LNOUA946-10	CLV104110	French Guiana	HQ571747
*Philonome lambdagrapha*	LNOUA928-10	CLV102310	French Guiana	HQ571730
*Philonome kawakitai*	GRANO044-11	AK0044	French Guiana	HQ571758
*Philonome* sp.	LNOUA958-10	CLV105310	French Guiana	KM224529

Details on the date and site of collection for each specimen, as well as a photograph are available through the DOI (http://dx.doi.org/10.5883/DS-PHILONO). The same DOI provides access to the sequence records and GenBank accession numbers (Table 1).

## Systematic accounts

### Philonome

Taxon classificationAnimaliaLepidopteraLyonetiidae

Chambers

Philonome Chambers, 1874: 96; Dyar 1903: 563; McDunnough 1939: 100; Davis 1983: 8; 1984: 25.
Philonome Type species: *Philonome clemensella* Chambers, 1874, by monotypy.
Phillonome [sic]: Chambers 1880: 196, 199. Incorrect subsequent spelling.
Phyllonome [sic]: Chambers 1882: 15. Incorrect subsequent spelling.

#### Adult.

*Head* (Fig. 18): Vestiture of vertex rough with piliform scales; frons smooth with broad, flat, appressed scales; a band of broad, spatulate scales between the bases of the antennae, along the transfrontal suture, bounded both above and below by piliform scales. Antenna filiform in both sexes; antennal pecten absent; scape elongate, ~2.2–2.4× length of adjacent pedicel. Labial palpus without bristle-like setae; 2^nd^ segment 2× longer than 1^st^, as long as 3^rd^. Maxillary palpus 5-segmented, longer than labial palpus. Proboscis naked, shorter than maxillary palpus.

**Figures 2–9.**
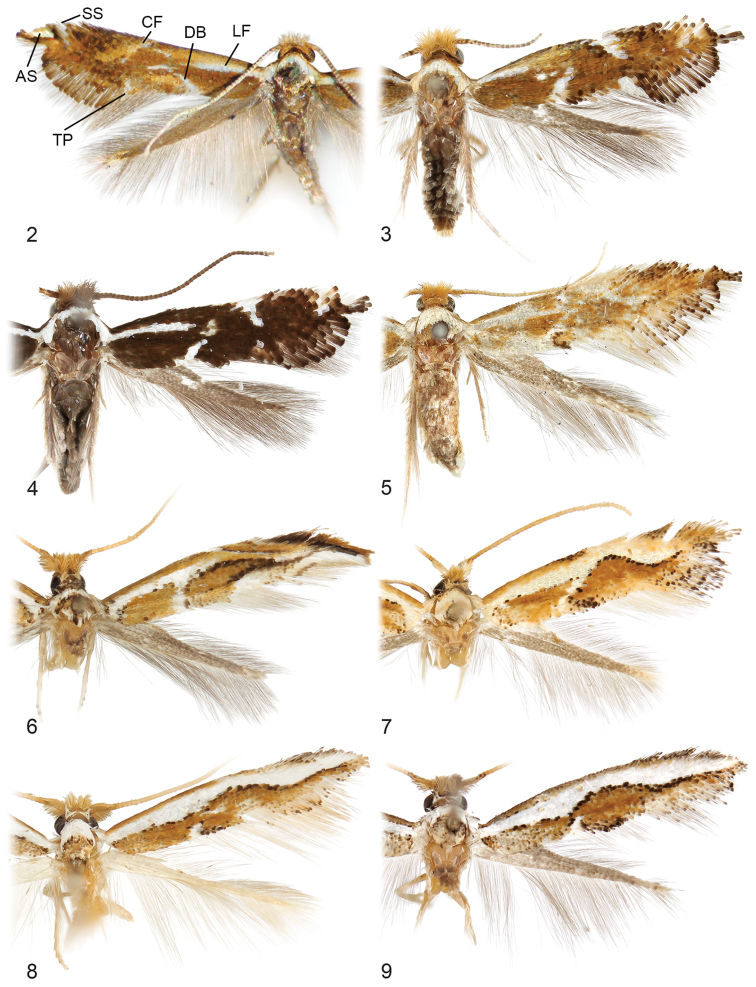
Adults. **2** *Philonome cuprescens*, ♂ (3.1 mm), abbreviations: AS = apical spot; CF = costal fascia; DB = dorsal bar; LF = longitudinal fascia; SS = subapical spot; TP = tornal patch **3** *Philonome wielgusi*, ♂ holotype (2.8 mm) **4** *Philonome nigrescens*, ♂ holotype (2.8 mm) **5** *Philonome clemensella*, ♂ (4.0 mm) **6** *Philonome lambdagrapha*, ♂ holotype (3.0 mm) **7** *Philonome curvilineata*, ♂ holotype (2.8 mm) **8** *Philonome euryarga*, ♂ (2.7 mm) **9** *Philonome albivittata*, ♂ holotype (2.8 mm). (Forewing lengths in parentheses).

*Thorax*: Foreleg epiphysis slender. Midfemur with apical tuft of elongate scales. Hind-tibia hairy dorsally. Forewing pattern elements (Fig. 2) including longitudinal fascia, costal fascia (absent in *Philonome euryarga*, *Philonome albivittata*, and *Philonome spectata*), subapical spot (present only in *Philonome cuprescens*, *Philonome wielgusi*, *Philonome nigrescens*, *Philonome clemensella*, and *Philonome lambdagrapha*), apical spot (present only in *Philonome cuprescens*, *Philonome wielgusi*, *Philonome nigrescens*, and *Philonome clemensella*), tornal patch, and dorsal bar (absent in *Philonome spectata*). Forewing venation (Fig. 20) with Rs 4-branched, all terminating on costa and arising from weak vein leading to M_1_; M_2_ and M_3_ stalked; CuA as one branch. Hindwing venation (Fig. 20) with Sc terminating on basal 1/4 of costa; Rs, M_2+3_, CuA weak, arising from weak vein leading to M_1_; CuP and 1A+2A weak, stalked.

**Figures 10–17.**
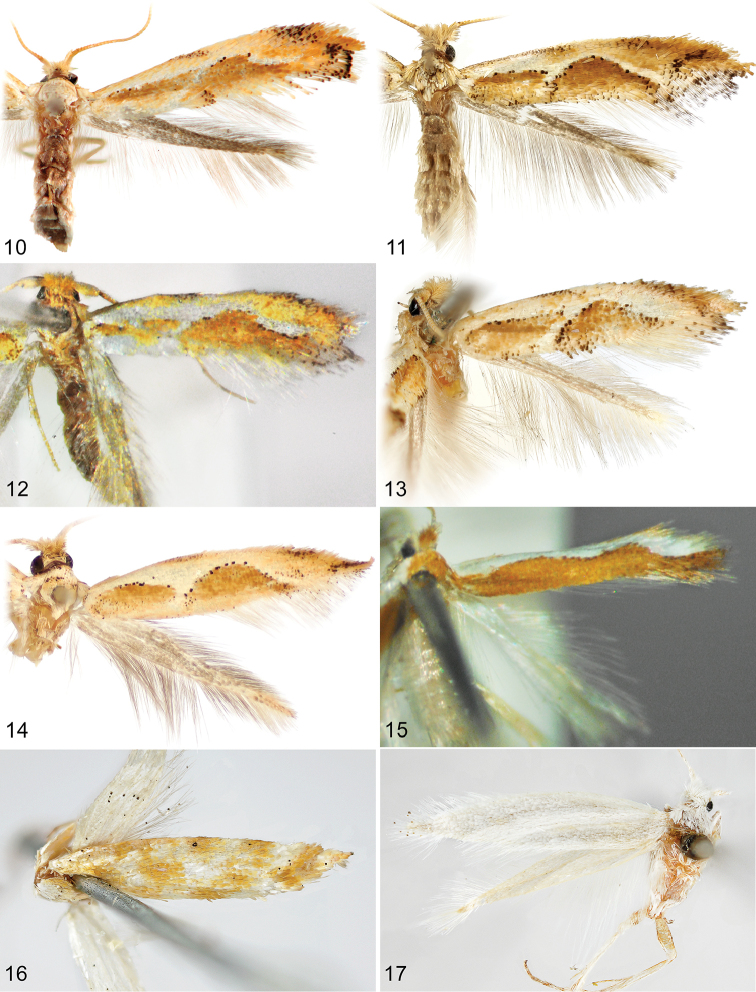
Adults. **10** *Philonome penerivifera*, ♀ paratype (3.6 mm) **11** *Philonome kawakitai*, ♀ holotype (3.8 mm) **12** *Philonome rivifera*, ♂ lectotype (3.4 mm) **13** *Philonome rivifera*, ♂ paralectotype (2.8 mm) **14** *Philonome* sp., CLV105310 (4.1 mm) **15** *Philonome spectata*, ♀ holotype (2.3 mm) **16** *Argyresthia luteella*, ♀ holotype (3.4 mm) **17** *Elachista dasycara* (= *Eurynome albella*),♀ holotype (4.0 mm). (Forewing lengths in parentheses).

**Figures 18–20.**
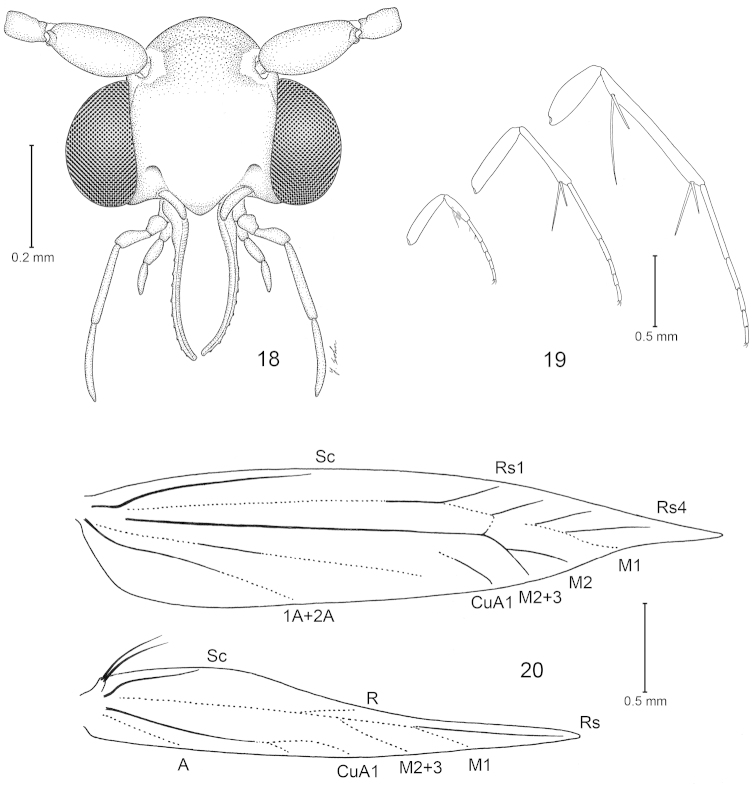
*Philonome clemensella*, body morphology. **18** Head, frontal view **19** Legs **20** Wing venation.

*Abdomen*: Coremata on male sternum VIII present posterolaterally, short and stiff (*Philonome albivittata* sp. n., *Philonome clemensella*, *Philonome euryarga*, and *Philonome wielgusi* sp. n.), long and hair-like (*Philonome lambdagrapha* sp. n.) or absent (*Philonome curvilineata* sp. n. and *Philonome rivifera*).

*Male genitalia*: Paired processes (uncus, Fig. 28) from tergum IX (tegumen) and often surrounding tuba analis either present or absent (*Philonome albivittata* sp. n. and *Philonome euryarga*); valva divided or deeply cleaved into two portions (*Philonome albivittata* sp. n., *Philonome clemensella*, *Philonome euryarga*, *Philonome penerivifera* sp. n., and *Philonome rivifera*) or entire; anellus funnel-shaped; basal ring of anellus moderately sclerotized; vinculum broad; saccus present.

**Figures 21–30.**
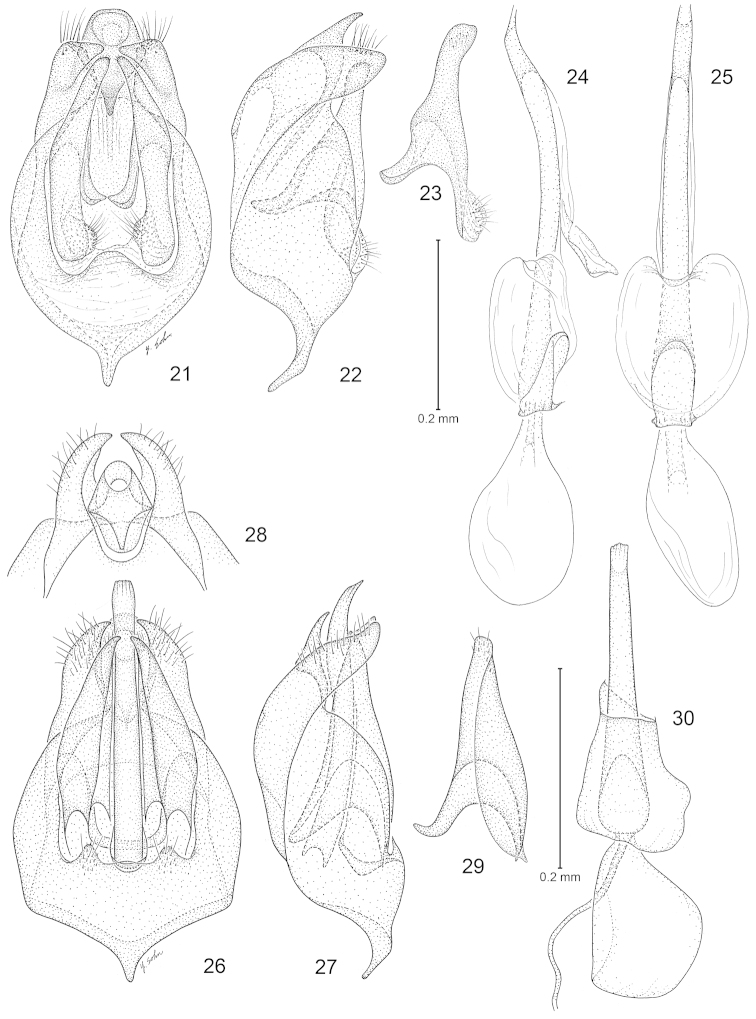
*Philonome*, male genitalia. **21**–**25** *Philonome cuprescens*. **21** Ventral view of genital capsule **22** Lateral view **23** Lateral view of valva **24** phallus, lateral view **25** phallus, ventral view **26**–**30** *Philonome wielgusi*, male genitalia. **26** Ventral view of genital capsule **27** Lateral view **28** Ventral view of anellus and uncus **29** Lateral view of valva **30** Phallus. (Scale lengths in parentheses).

*Female genitalia*: Ovipositor telescopic with two primary segments; papillae anales semi-elliptical, setose; lamella antevaginalis conical or cylindrical; additional protrusion behind ostium bursae present (*Philonome nigrescens* and *Philonome rivifera*) or absent; ductus bursae slender; corpus bursae obovate or elliptical; signum absent.

#### Biology.

Chambers (1878) mentioned that he repeatedly collected *Philonome clemensella* from the type locality where *Gleditschia triacanthos* L., *Ulmus americana* L., *Prunus serotina* Ehrh., and *Celtis occidentalis* L. grow in the immediate vicinity. He then assumed that the larvae may feed on some weeds or shrubs growing nearby. Forbes (1923) noted that the larvae of *Philonome clemensella* feed on hickory and linden. These records were, however, based on ambiguous label data which state only plant names without details and thus require verification. Nothing is known about the biology for other congeners of *Philonome clemensella*.

Included species (arranged by the similarities in the forewing pattern and the male genitalia):

*Philonome cuprescens* Walsingham, 1914

*Philonome wielgusi* sp. n.

*Philonome nigrescens* sp. n.

*Philonome clemensella* Chambers, 1874

*Philonome lambdagrapha* sp. n.

*Philonome curvilineata* sp. n.

*Philonome euryarga* Meyrick, 1915

*Philonome albivittata* sp. n.

*Philonome penerivifera* sp. n.

*Philonome kawakitai* sp. n.

*Philonome rivifera* Meyrick, 1915

*Philonome spectata* Meyrick, 1920

*Philonome* sp.

#### Keys to the species of *Philonome* based on external appearance

**Table:** 

1	Forewing with longitudinal fascia extending near apex	**2**
–	Forewing with longitudinal fascia not extending beyond discal cell	**5**
2	Forewing ground color brown or reddish brown	**3**
–	Forewing ground color black	***nigrescens* sp. n.**
3	Forewing with costal fascia curved	**4**
–	Forewing with costal fascia straight	***wielgusi* sp. n.**
4	Yellow lining along costal fascia of forewing narrow	***clemensella* Chambers**
–	Yellow lining along costal fascia of forewing broad	***cuprescens* Walsingham**
5	Forewing with dorsal bar	**6**
–	Forewing without dorsal bar	***spectata* Meyrick**
6	Forewing with dorsal bar connected with longitudinal fascia	**7**
–	Forewing with dorsal bar separated from longitudinal fascia	**8**
7	Fore- and hindwing fringes pale grayish orange	***euryarga* Meyrick**
–	Fore- and hindwing fringes brownish gray	***albivittata* sp. n.**
8	Forewing with costal fascia	***lambdagrapha* sp. n.**
–	Forewing without costal fascia	*rivifera* Meyrick and allied species*

* Four species, *curvilineata* sp. n., *rivifera* Meyrick, *penerivifera* sp. n., and *kawakitai* sp. n., are indistinguishable from one another based on external appearance; see Table 2 for their differences in male genitalia (except *kawakitai* sp. n. whose males are unknown).

**Table 2.** Comparison of three similar species of *Philonome*, *Philonome curvilineata*, *Philonome penerivifera*, and *Philonome rivifera* in the male genitalia.

Characters	*curvilineata*	*penerivifera*	*rivifera*
Apical area of valva	entire	emarginated	bifid
Short spiniform setae on cucullus	present	absent	absent
Saccular portion of valva	not separate	separate	separate
Lateral area of vinculum	subtruncate	strongly protruding	slightly protruding
Saccus	1/4 as long as valva	3/5 as long as valva	1/2 as long as valva

### Philonome cuprescens

Taxon classificationAnimaliaLepidopteraLyonetiidae

Walsingham, 1914

Figs 2 , 21–25

Philonome cuprescens Walsingham, 1914: 346; Davis 1984: 25.

#### Adult

(Fig. 2). *Head*: Vertex orange; space between antennal scapes lined with broad pale orange scales; frons brownish white with luster. Antenna 5/6 as long as forewing; scape orange dorsally, brownish white ventrally, pecten reddish brown; flagellomere dark brown on distal half, pale brownish gray on basal half. Labial palpus 1/4 as long as maxillary palpus, pale orange, pale orange. Maxillary palpus lustrous yellowish white dorsally, gray ventrally.

*Thorax*: Patagium orange; tegula white, tinged with brown basally, yellowish brown subbasally; mesonotum brown with white transverse band at anterior 1/5, lined with yellowish brown anteriorly. Foreleg with coxa, femur and tibia dark brown on exterior surface, lustrous pale reddish brown on interior surface; tarsomeres pale brown dorsally, pale orange ventrally. Midleg with coxa lustrous pale orange; femur lustrous pale orange, tinged with dark grayish brown apically; tibia dark brown dorsally, pale orange ventrally; tarsomeres pale reddish brown dorsally, pale orange ventrally. Hindleg with coxa pale orange; femur pale grayish brown, tinged with pale orange ventrobasally; tibia brown dorsally, pale orange ventrally, with mixture of pale orange and pale brown piliform scales of tuft ventrally; tarsomeres pale reddish brown dorsally, pale orange ventrally. Forewing length 2.8–3.9 mm (n = 2), brown, intermixed with dark brown scales in postmedian area; longitudinal fascia white, closer to costa than to dorsum, accompanied with yellowish brown fascia anteriorly; costal fascia yellowish brown, curved to apex at the middle, accompanied with narrow, white line along lower margin in costal 1/2; dorsal bar white, curved in terminal 1/4, accompanied with yellowish brown spreading in dorsal area, almost connected with longitudinal fascia; subapical spot white, narrow, curved; apical spot white, suffused with reddish brown costally; tornal patch very small; fringe elongate scales dark brown, hairy scales dark grayish brown. Hindwing and fringe dark grayish brown.

*Abdomen*: Terga lustrous, dark reddish brown; sterna lustrous, pale yellow.

*Male genitalia* (Figs 21–25): Tegumen rectangular, with subtrapezoidal protrusion apically and subtriangular process laterally; apical protrusion 1/2 as long as valva, with round depression ventro-subapically and triangular anterior extension. Valva elongate, digitate on distal half, setose subapically; costa convex at basal 2/5; sacculus as small, setose bulge. Vinculum broad, elliptical, anterior margin convex medially; saccus short, narrow-subtriangular. Phallus slightly curved at distal 1/3, of even width on distal 3/4, broadened on basal 1/4.

Female unknown.

#### Types.

Holotype: male, “Type” [circular label with red borders], “Amula, 6000ft. GUERRERO MEXICO VIII 18 (H.H.Smith) (Gdm. Slvn) 66776”, “Walsingham Collection, 1910-427”, “Philonome ♂ cuprescens Wlsm. Biol. C. Am. Lep. Het. 4. p346, 1914 TYPE ♂ descr” [label with black marginal lines], BMNH. Paratypes: Same data as holotype: 1♂, 1 ex. [hindwing & abdomen missing], Type no. 66778 & 66779, [GSN] USNM 34210 (♂), USNM.

#### Material examined.

**Mexico:** Same locality as holotype: 1♂, 2 ex., 18 September [no year] (HH Smith), BMNH.

#### Distribution.

Mexico (Guerrero).

### Philonome wielgusi

Taxon classificationAnimaliaLepidopteraLyonetiidae

Sohn & Davis sp. n.

http://zoobank.org/EDA70A5B-C1CD-4432-A242-EB7D19E71501

Figs 3 , 26–30

#### Diagnosis.

This species is similar to *Philonome clemensella* in external appearance but can be distinguished from the latter in having shorter longitudinal fascia and straight costal fascia on the forewing. In the male genitalia, the lateral processes on the uncus are larger in *Philonome wielgusi* than in *Philonome clemensella* and the valvae are not divided at all in *Philonome wielgusi*.

#### Adult

(Fig. 3). *Head*: Vertex yellowish brown, paler to frons; frons lustrous, pale yellowish gray. Antenna 2/3 as long as forewing; scape dark brown dorsally, with broad, lustrous, pale yellowish gray patch ventrally; first six flagellomeres dark brown dorsally, narrowly white ventrally; the remaining flagellomeres entirely dark brown. Labial palus straight, very small, 3/5 as long as antennal scape, pale orange, apex obtuse. Maxillary palpus 2× longer than labial palpus, yellowish gray; each segment tinged with pale orange apically.

*Thorax*: Patagium yellowish brown. Tegula dark yellowish brown on basal 1/4, pale grayish brown on distal 3/4, paler distad. Mesonotum lustrous white on anterior 1/3, dark grayish brown on posterior 2/3. Foreleg with coxa dark grayish brown laterally, silvery gray mesally; femur and tibia dark grayish brown, paler ventrad; tarsomere I to II pale orange, with narrow dark brownish gray patch dorsally, the remaining tarsomeres entirely dark grayish brown. Midleg with coxa and tibia dark grayish brown laterally, lustrous pale orange mesally; tibia dark brownish gray dorsally, lustrous pale orange ventrally; tarsi dark brownish gray. Hindleg with coxa and femur lustrous, pale orange; tibia lustrous, yellowish gray, sparsely hairy dorsally, with dense spiniform setae ventrally; tarsi dark brownish gray. Forewing length 2.3–3.0 mm (n = 8), brown, dorsum dark brownish gray basally; longitudinal fascia, white, extending from base to basal 1/3 of forewing, costal fascia white, at distal 2/5 of costa, oblique, adjacent to a slender dark brown line on anterior side; subapical and apical spots white; tornal patch white, triangular; dorsal bar white, oblique; fringe dark orange, each scale with dark brown tip. Hindwing and fringe gray.

*Abdomen*: Terga lustrous, gray; sterna lustrous, pale orange. Tergum VIII of male rectangular; sternum VIII subrectangular, broadly emarginated posteriorly, with oblique furrow laterally and short, stiff coremata posterolaterally.

*Male genitalia* (Figs 26–30): Tegumen subtrapezoidal, with semi-elliptical protrusion apically and falcate processes (uncus) laterally; apical protrusion with round opening dorsoposteriorly, connected with U-shaped groove basally; teguminal process 1/2 as long as valve. Valva elongate, broadened to base; apex obtuse, sparsely setose; sacculus broadened at basal 1/3, narrowed distally, nearly as long as valva. Anellus extending to basal 3/5 of phallus; juxta forming a ridge connected to anellus. Vinculum broad, smoothly angulate laterally, broadly triangular anteriorly, with setose bulge near base of valva; saccus short, subtriangular. Phallus narrowed to apex, slightly curved at middle.

Female unknown.

#### Types.

Holotype: male, “ARIZONA: Cochise Co.: Sierra Vista 5131 Bannock 2 IX 1988”, “Attracted to (E, Z) – 3 13 ODDOH @ 1615-1730 hrs.”, “R. S. Wielgus Collector”, USNM. Paratypes (78♂): **USA:** Arizona: Cochise Co.: Chiricahua Mountains., Sunny Flat Campground: 1♂, 28 July 1989, B & JF Landry, CNC. Sierra Vista: 5131 Bannock, 10♂, 31 August 1988 (RS Wielgus), on pheromone trap; 18♂, 1 September 1988, [GSN] USNM 31056; 12♂, 2 September 1988, [GSN] USNM 29950; 2♂, 5 September 1988; 1♂, 6 September 1988; 7♂, 9 September 1988; 1♂, 10 September 1988; 1♂, 14 September 1988; 3♂, 15 September 1988; 8♂, 16 September 1988; 4♂, 17 September 1988; 7♂, 18 September 1988, USNM. Graham Co.: Pinaleno Mountains: Wet Canyon: 3♂, 22 July 1989, B & JF Landry, CNC.

#### Distribution.

Southwestern United States (Arizona).

#### Etymology.

The species name is a patronym in honor of Mr. Ronald S. Wielgus, who collected nearly the entire type series.

#### Remarks.

As reported by the collector, Ronald Wielgus, and indicated on specimen labels, nearly all moths were collected in the late afternoon, between 16:15 and 17:30 hours. All 157 adults collected thus far are males.

### Philonome nigrescens

Taxon classificationAnimaliaLepidopteraLyonetiidae

Sohn & Davis sp. n.

http://zoobank.org/8CF6C86B-3C26-48E6-B7F6-1366DC45017B

**Figures 31–36.**
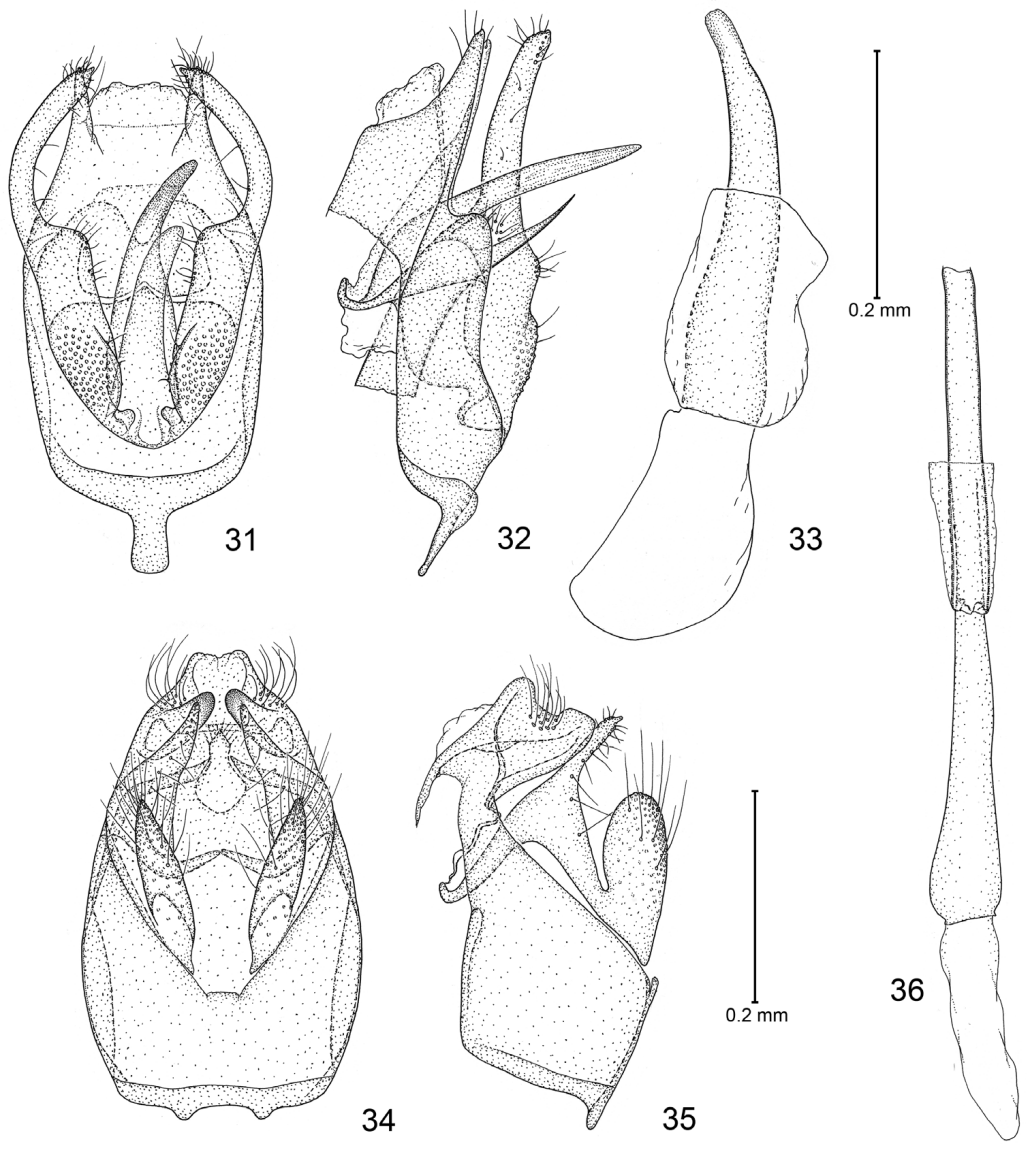
*Philonome*, male genitalia. **31**–**33** *Philonome nigrescens* **31** Ventral view of genital capsule **32** Lateral view **33** Phallus **34**–**36** *Philonome clemensella*. **34** Ventral view of genital capsule **35** Lateral view **36** Phallus.

Figs 4 , 31–33 , 57–58

#### Diagnosis.

This species is easily distinguished from all other congeners in possessing a black ground-color of the forewing and an elongate process on the transtila of the male genitalia.

#### Adult

(Fig. 4). *Head*: Scales on vertex dark reddish brown, as long as antennal scape, directed forward; semicircular, dome-like scale cap on anterior vertex between antennal scapes, slightly concave anteriomedially, lustrous, pale yellowish gray; frons lustrous, yellowish gray. Antenna 3/5 as long as forewing; scape dark brown dorsally, reddish brown laterally, pale brownish gray ventrally, with flabellate, pale brownish gray scape cap anteroventrally and pecten; flagellomeres dark brown dorsally, pale brownish gray ventrally. Labial palpus straight, slender, conical, obtuse apically, small, 1/2 as long as antennal scape, lustrous, pale yellowish gray.

*Thorax*: Patagium white on distal half, dark brown on basal half; tegula white, tinged with dark brown basally; mesonotum dark brown with coppery luster. Foreleg with coxa to tarsomeres lustrous orange-white, narrowly tinged with gray dorsally. Midleg with coxa to tibia lustrous orange-white; femur with broad pale reddish brown patch dorsally; tibia broadly dark gray dorsally; tarsomeres dark gray, paler ventrally. Hindleg with coxa lustrous pale orange, tinged with brown basally; femur lustrous pale orange; tibia and tarsomeres dark gray dorsally, lustrous pale orange ventrally; tibia spinose dorsally, with hair tufts ventrally. Forewing length 2.1–3.2 mm (n = 7), dark brown with coppery luster; longitudinal fascia white, extending from base to basal 1/3 of forewing; costal fascia white, straight, broadened at costa; dorsal bar white; subapical, apical and tornal spots white; fringe gray. Hindwing gray, paler to base; fringe gray.

*Abdomen*: Terga lustrous, dark grayish brown; sterna lustrous, yellowish gray ventrally.

*Male genitalia* (Figs 31–33): Tegumen trapezoidal, with digitate process posterolaterally; teguminal process 1/3 as long as valva, sparsely setose on dorsoapical 1/2; tuba analis arising between teguminal processes. Valva subtrapezoidal on basal half, digitate on distal half, densely setose apically and at middle, sparsely setose on distal half; sacculus broadly swollen and granulate at basal 1/3, convex and setose at distal end. Transtilla with elongate process 3/4 as long as phallus. Juxta with semicircular bulge, connected to anellus.Vinculum broad, subquadrate; saccus quadrate, 1/2 as long as lateral process of tegumen. Phallus slightly curved, enlarged posteriorly; apex with linguiform carina.

*Female genitalia* (Figs 57–58): Apophyses posteriores 1.8× longer than apophyses anteriores. Lamella anteveginalis dome-shaped, slanted to ostium bursae. Sclerotized protrusion bearing ostium cylindrical, surrounded with conical membranous area. Ductus bursae as long as corpus bursae, narrow; inception of ductus seminalis at posterior 1/3 with a cylindrical sclerotization. Corpus bursae obovate.

#### Types.

Holotype: male, “ARIZONA: Cochise Co. Sierra Vista 5131 Bannock 18 IX 1988”, “R Wielgus Collector”, “Attracted to 1988 Farchan (Z, Z)-3, 13 ODDA@1620hrs. in pheromone trap” [hand-written], USNM. Paratypes (8♂, 1♀): **USA:** Arizona: Cochise Co.: Same locality as holotype: 1♂, 13 May 1988, attracted to pheromone trap; 3♂, 28 August 1988 (R S Wielgus), attracted to pheromone trap; 1♂, 17 September 1988, all USNM. Pima Co.: Station Catalina: 1♂, [“iss”] 13 June 1913 (Hopk.), “from trunk of evergreen Oak”, [GSN] USNM 16406, USNM. Santa Cruz Co.: Peña Blanca Campground: 2♂, 22 August 1988, attracted to pheromone trap, [WSN] USNM wing 29949, USNM. New Mexico: Grant Co.: Silver City: 1♀, 5 June 1974 (PM Jump), [GSN] USNM 34355, USNM.

#### Etymology.

The species name is derived from the Latin verb ‘nigrescere’ meaning “verging on black” and refers to the black ground color of the forewing of this species.

#### Distribution.

Southwestern United States (Arizona, New Mexico).

### Philonome clemensella

Taxon classificationAnimaliaLepidopteraLyonetiidae

Chambers, 1874

Figs 5 , 34–36 , 59–60

Philonome clemensella : Chambers 1874: 97; Dyar 1903: 563; McDunnough 1939: 100; Davis 1983: 8.
Philonome staintonella : Chambers 1876: 136. *Nomen nudum*

#### Adult

(Fig. 5). *Head*: Vertex convex medially; scales on vertex orange, as long as antennal scape, slanted forward; semicircular, dome-like scale cap on anterior vertex between antennal scapes with compact appressed, lustrous pale yellow scales; frons smooth, lustrous pale yellow. Antenna 3/5 as long as forewing; scape reddish brown dorsally, lustrous pale yellow ventrally, with fan-shaped scale cap anterioventrally; flagellomeres dark reddish brown dorsally, lustrous pale yellow ventrally. Labial palpus straight, slender, very small, 1/2 as long as antennal scape, pale orange, apex acuminate. Maxillary palpus 3× longer than labial palpus, pale orange.

*Thorax*: Patagium reddish brown; tegula pale orange-white, suffused with reddish brown basally; mesonotum pale orange-white. Foreleg with coxa lustrous orange-white, tinged with gray dorsobasally; femur and tibia dark brownish gray dorsally, lustrous orange-white ventrally; tarsi orange-white, lustrous ventrally. Midleg with coxa lustrous orange-white; femur to tarsi pale orange dorsally, lustrous orange-white. Hindleg with coxa to tarsi lustrous orange-white; femur narrowly tinged with pale orange dorsally; tibia spinose dorsally, with hair-tufts ventrally. Forewing length 2.8–4.4 mm (n = 70), reddish brown; longitudinal fascia, white from base to the middle of forewing, often connected with white dorsal bar; costal fascia at distal 1/3 of costa, white, terminal 1/3 curved to apex, accompanying a row of dark brown scales caudad; subapical spot orange-white; tornal patch white, semicircular, borders blurred; elongate scales on apex, terminal 1/3 of costa, termen, with dark brown tips; fringe orange-white. Hindwing and fringe lustrous pale gray.

*Abdomen*: Terga lustrous, yellowish gray; sterna lustrous orange-white. Male tergum VIII trapezoidal; male sternum VIII subrectangular, broadly emarginated posteriorly, with oblique furrow laterally and short, stiff coremata posterolaterally.

*Male genitalia* (Figs 34–36): Tegumen trapezoidal, with subrectangular protrusion apically, strongly sclerotized, digitate process laterally, and short, lanceolate sclerite at center; long setae above teguminal process; tuba analis arising from dorsal area of apical protrusion. Valva deeply divided into two portions; costal portion triangular on basal half, elongate on distal half, sparsely setose, apex protruding; saccular portion elliptical, more densely setose to apex. Anellus extending to middle of phallus. Vinculum broadly sclerotized, capsulate, with a pair of small protrusions on distal margin. Phallus of even width except slightly-swollen at basal 1/6, slightly bifid apically.

*Female genitalia* (Figs 59–60): Apophyses posteriores 2× longer than apophyses anteriores. Elongate scales on posteroventral margin of A8. Lamella antevaginalis short, cylindrical, with ridge posterolaterally. Ductus bursae as long as corpus bursae, narrow on anterior 1/2; inception of ductus seminalis at middle of ductus bursae, bulged, with a sclerotized ring. Corpus bursae obovate.

#### Types.

Lectotype (designated here): male, “48” [hand-written], “Type No. 522 U.S.N.M.” [red label], “*Philonome clemmensella* [sic] K[entuck]y. 5961Lis1 Cham[bers]” [hand-written], USNM. Paralectotypes: **USA:** Kentucky: 4♀, 3 ex., June 14 [no year] (Chambers), Type no. 1311, MCZ.

#### Material examined.

**Canada:** Ontario: Ottawa-Careton, Dunrobin: 1♀, 9 July 2007, CNCLEP00035968; 28 July, 2007, CNCLEP00038457, (L Scott). Quebec: 2♂, 21 July 2004, CNCLEP00006545, (JF Landry), CNC. **USA:** Alabama: Monroe Co.: Haines Island Park (31°43'23"N, 87°28'10"W): 2♂, 2♀, 26–27 May 1995 (R Brown, J MacGown & D Pollock), MSU. District of Columbia: Unspecified locality: 1♂, no date (Fernald); 1♂, 28 June 1885 (Fernald), “on oak”; 1♀, 21 June 1886 (Fernald); 1♂, 11 July 1896, “Hickory”; 3♀, 2–4 June 1897, “from Linden”; 1♀, September 1953, USNM. Florida: Pinellas Co.: Dunedin: Hammock Park: 1♂, 22 April 1987 (LC Dow), [GSN] USNM 96414, USNM. Kentucky: No specified locality: 1♂, no date & collector, USNM. Illinois: Macon Co.: Decatur: 1♂, 8–15 June [no year], USNM. Putnam Co.: 1♀, 30 June 1976 (MO Glenn), USNM. Maryland: Montgomery Co.: Takoma Park: 1♂, 1♀, 8 July 1986 (WE Steiner); 1♂, 7 July 1986, USNM. Wicomico Co.: 1km SW Sharptown at Plum Creek: 1♀, 12 July 1986 (JM Hill et al.), USNM. Massachusetts: Dukes Co.: Martha’s Vineyard: 1♂, 13 July [no year] (FM Jones); 1 ex., 29 July [no year], all USNM. New Jersey: Burlington Co.: Medford: Lake Pine: 1♂, 13 July 1974 (DC Rentz), USNM. Essex Co.: Caldwell: 3♂, 2♀, 8 July 1900 (WD Kearfott), USNM. Essex County Park: 1♀, 20 May [no year] (WD Kearfott); 1♂, 7 July [no year], GSN: USNM 29977; 1♂, 12 July 1901; 1♂, 15 July [no year], USNM. Montclair: 1♀, 10 July [no year] (WD Kearfott), USNM; 1♂, 18 July [no year] (WD Kearfott), USNM. New York: Tompkins Co.: Ithaca: Six Mile Creek: 1♀, 23 July 1960 (RW Hodges), USNM. Unspecified locality: 1♀, “4971/ WLSM. 1906” (Beutenmueller), USNM. North Carolina: Craven Co.: Cherrypoint: 1♀, 3 July 1961 (SS Nicolay); 1♀, 12 July 1961; 1♀, 21 July 1961, all USNM. Harnett Co.: Spout Springs: 1♂, 25 August 1984 (WE Steiner et al.), USNM. Ohio: Hamilton Co.: Cincinnati, 1 ex., 23 June 1906 (A Braun); 1♂, 1♀, 27–28 June 1906, [GSN] USNM 16405 (♂); 1♀, 24 July 1907; 1♂, 3 August 1907; 1♂, 16 June 1908, all USNM. PENNSYLVANIA: Adams Co.: Arendtsville: 5♂, 6 July 1921 (SW Frost), USNM, GSN: USNM 29575. Allegheny Co.: Oak Station: 1♂, 6 July 1907 (F Marloff); 1♂, 11 July 1907, all USNM. Beaver Co.: New Brighton: 2♂, 11 July 1907 (Merrick Museum); 1♂, 23 July 1907; 1♂, 2♀, 26 July 1907, [GSN] USNM 34213 (♀), USNM. South Carolina: Charleston Co.: McClellanville: Wedge Plantation: 1♀, 11 May 1981 (RW Hodges), USNM, GSN: USNM 34212. Tennessee: Cocke Co.: Great Smoky Mt. National Park: Foothills Parkway (35°48'59"N, 83°13'11"W): 3♂, 1♀, 9 June 2002 (RL Brown & SM Lee), MSU. Texas: Harris Co.: 1♂, 20 May 1984 (Bellaire), [GSN] USNM 96415; 1♂, 2 April 1986, all USNM. Virginia: Fairfax Co.: 1km E Fairfax City: 1♂, 9 July 2005 (J Brown), USNM. Unspecified locality: 1♀, 27 June 1886, USNM. West Virginia: Morgan Co.: Sleepy Creek Forest: 2♂, 1 July 2010 (J Glaser); 1♂, 16 July 2011; 1♂, 19 July 2011; 1♂, 21 July 2011, USNM.

#### Distribution.

Eastern Canada and the United States west to Texas.

#### Host plants.

Hickory (Juglandaceae: *Carya*) and linden (Tiliaceae: possibly *Tilia americana* L.) (Forbes 1923). These are from the label data in the USNM collection. The collection also includes a specimen whose label data states that it came from oak (Fagaceae: *Quercus*). The label data give no details other than plant common names. Therefore, it is not clear if these records refer to larval host plants or where the adults were collected.

### Philonome lambdagrapha

Taxon classificationAnimaliaLepidopteraLyonetiidae

Sohn, Davis & Lopez-Vaamonde sp. n.

http://zoobank.org/E4E1964A-D664-4342-B555-DBC16C934821

Figs 6 , 37–39

#### Diagnosis.

This species is similar to *Philonome curvilineata* in external appearance but differs from the latter in having the longitudinal and costal fasciae separate (continuous in *Philonome curvilineata* and larger apical protrusion on the tegumen in the male genitalia.

#### Adult

(Fig. 6). *Head*: Vertex orange on posterior 2/3, pale orange on anterior 1/3; scales on dorsum of occiput dark grayish brown; scales between antennal scapes lustrous pale orange; frons lustrous pale yellow. Antenna 4/5 as long as forewing; scape as long as diameter of eye, lustrous orange dorsally, lustrous pale yellowish gray laterally and ventrally, lustrous pale grayish brown apically; flagellomeres pale orange dorsally, lustrous pale yellow ventrally. Labial palpus 1/2 as long as antennal scape, lustrous pale yellowish gray.

*Thorax*: Patagium dark brown; tegula lustrous pale yellow, intermixed with orange scales basally; mesonotum silvery white with dark brown transverse band along anterior and posterior margins and at anterior 1/3, with orange transverse band at middle. Foreleg with coxa lustrous pale yellow; femur lustrous pale yellow, intermixed with pale grayish brown laterally; tibia dark brown dorsally, pale grayish yellow ventrally; tarsomeres dark brown dorsally, pale orange ventrally. Midleg with coxa and femur lustrous pale yellow; tibia and tarsomeres grayish brown dorsally, pale yellow ventrally. Hindleg consumed for DNA extraction. Forewing length 3.0 mm (n = 1), reddish brown; costa black in distal 1/3; longitudinal fascia extending to apical streak, straight, white on basal 1/2, juxtaposed with a slender, intermittent black line along lower border, sinuous, black on distal 1/2; costal fasciae slender, extending to apex; subapical streak white, juxtaposed with slender black line along lower border; apical streak white, connected with longitudinal fascia; dorsal bar white, juxtaposed with black along outer border; dorsal margin sparsely irrorated with black scales on basal 1/6 and at middle; tornal patch elongate, white, juxtaposed with black along upper border, irrorated with dark brown scales along outer border; marginal streak dark brown; fringe brown on distal 1/3 of costa, pale yellowish gray along termen. Hindwing brownish gray; fringe pale grayish brown.

*Abdomen*: Male tergum VIII and sternum VIII subquadrate; coremata piliform, as long as tergum VIII.

**Figures 37–46.**
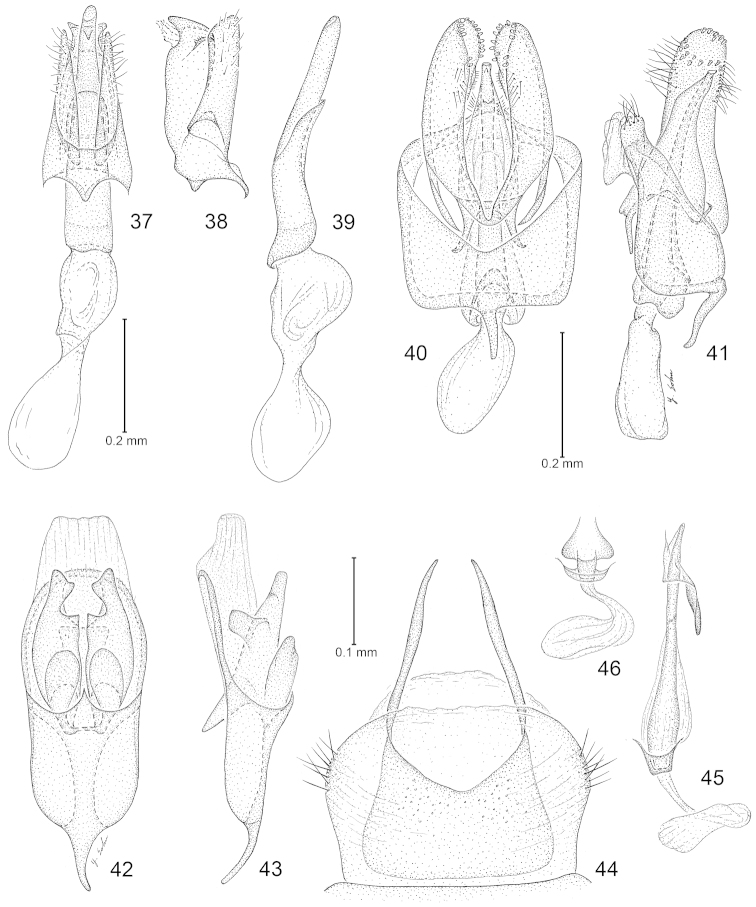
*Philonome*, male genitalia. **37**–**39** *Philonome lambdagrapha* **37** Ventral view of genital capsule and phallus **38** Lateral view **39** Phallus **40**–**41** *Philonome curvilineata*. **40** Ventral view of genital capsule and phallus **41** Lateral view **42**–**46** *Philonome euryarga* **42** Ventral view of genital capsule **43** Ventral view **44** abdominal tergum VIII (caudal end directed upward) **45** Lateral view of phallus **46** Anterior base of phallus, ventral view.

*Male genitalia* (Figs 37–39): Tegumen nearly as long as valva, semi-elliptical on basal 3/4, rectangular on distal 1/4, with small lateral protrusion dorsoposteriorly. Valva elongate, lobate, sparsely setose on outer surface. Juxta liguiform, 1/2 as long as valva. Vinculum broad, gradually broadened anteriorly, with medial and lateral protrusions along anterior margin; Phallus slightly curved at basal 2/5, broadened posteriorly.

Female unknown.

#### Type.

Holotype: male, “ID#: CLV102310 [red letters] French Guiana: [Régina, Nouragues Research Station] [Lt:4.1 Ln:52/] Carlos Lopez Vaamonde 23-Jan-2010 DNA Barcode LNOUA928-10 [green letters in blue row]”, “Nou68”, “Genitalia slide DRD ♂ USNM 34621” [green label], USNM.

#### Distribution.

French Guiana.

#### Etymology.

The species name is derived from the Greek letter ‘lambda’ and a suffix derived from the Greek ‘graphein’ meaning “to write”, and refers to the white fascia of the forewing resembling a lambda (λ).

### Philonome curvilineata

Taxon classificationAnimaliaLepidopteraLyonetiidae

Sohn, Davis & Lopez-Vaamonde sp. n.

http://zoobank.org/992460F0-1A1B-4054-8818-2A32A56B77B6

Figs 7 , 40–41

#### Diagnosis.

This species is indistinguishable from *Philonome rivifera* Meyrick in external appearance but differs from the latter in having the apex of the valva in the male genitalia entire (*vs.* bifid in *Philonome rivifera*).

#### Adult

(Fig. 7). *Head*: Scales of vertex orange on posterior 2/3, pale orange on anterior 1/3; scales on dorsum of occiput dark brown, orange on basal 1/4; scales between antennal scapes lustrous pale orange; frons very small, pale orange. Antenna 4/5 as long as forewing; scape as long as diameter of eye, pale orange, paler ventrad; flagellomeres pale orange dorsally, silvery white ventrally. Labial palpus 1/2 as long as antennal scape, lustrous pale orange.

*Thorax*: Scales of patagium orange with dark brown tips; tegula pale orange, intermixed with orange scales basally; mesonotum lustrous orange white, with pale orange transverse band along anterior and posterior margins and at middle. Foreleg with coxa and femur lustrous pale orange; tibia orange, intermixed with dark brown scales dorsally, orange white ventrally; tarsomeres orange dorsally, pale orange ventrally; first tarsomere sparsely intermixed with dark brown scales dorsally. Midleg with coxa and femur lustrous pale orange; tibia brownish orange dorsally, lustrous orange white ventrally; tarsomeres pale orange dorsally, pale yellow ventrally. Hindleg consumed for DNA extraction. Forewing length 2.8 mm (n = 1), reddish brown, slightly paler along dorsal area; costal area yellowish brown on basal 1/2, brownish white above the curvature of longitudinal fascia, pale orange on distal 1/4, intermixed with black scales on middle and distal 1/4 of costa; longitudinal fascia continuous to near apex; convex at distal 1/3, white, juxtaposed with slender black line along lower border; dorsal bar straight, white, juxtaposed with slender, intermittent, black line along outer border; black irroration at middle of dorsal margin and on tornal area; fringe orange on distal costa and apex; scales of fringe along termen pale yellowish gray on basal 2/3, black on distal 1/3. Hindwing brownish gray; fringe pale grayish brown.

*Abdomen*: Male tergum VIII rectangular; male sternum VIII rectangular, broadly emarginated posteriorly.

*Male genitalia* (Figs 40–41): Tegumen rectangular, convex posteriorly, with sparsely setose, small bulge apically; tuba analis arising from dorsoposterior region of tegumen. Valva digitate, slightly enlarged on basal 1/2, flattened apically, with stout spiniform setae along edges of apical area and with piliform setae in inner surface of costal and saccular areas. Anellus conical, nearly as long as phallus. Vinculum broad, rectangular; saccus elongate, 1/4 as long as valva. Phallus slightly curved at distal 1/5, narrowing to apex, greatly broadened in basal 1/6.

Female unknown.

#### Type.

Holotype: male, “ID#: CLV68110 [red letters] French Guiana: [Régina, Nouragues Research Station] [Lt:4.1 Ln:52/] Carlos Lopez Vaamonde 20-Jan-2010 DNA Barcode LNOUA586-10 [green letters in blue row]”, “Nou37”, “Genitalia slide DRD ♂ USNM 34620” [green label], USNM.

#### Distribution.

French Guiana.

#### Etymology.

The species name, an adjective, is derived from the Latin words ‘curvus’ and ‘lineatus’, together meaning “curved line” and refers to the curved longitudinal fascia on the forewing of this new species.

### Philonome euryarga

Taxon classificationAnimaliaLepidopteraLyonetiidae

Meyrick, 1915

Figs 8 , 42–46

Philonome euryarga Meyrick, 1915: 250.

#### Adult

(Fig. 8). *Head*: Vertex reddish orange on posterior 2/3, pale orange on anterior 1/3; scales on interspace between antennal scapes yellowish white; frons lustrous, yellowish white; occipital area white. Antenna 3/5 as long as forewing; scape pale orange dorsally, pale yellowish gray; flagellomeres pale reddish orange dorsally, yellowish white ventrally. Labial palpus as long as maxillary palpus, lustrous, pale yellowish gray, intermixed with dark brown scales apically. Maxillary palpus lustrous yellowish white.

*Thorax*: Patagium and tegulae white; mesonotum white in anterior 1/2, reddish brown in posterior 1/2, with a dark brown transverse band medially; mesoscutellum brownish gray. Foreleg lustrous yellowish white, with narrow brownish gray area dorsally. Midleg reddish orange dorsally, lustrous yellowish white ventrally. Hindleg pale orange dorsally, lustrous yellowish gray ventrally. Forewing length 2.7 mm (n=1), reddish brown; costa brown; longitudinal fascia white, spanning entire costal area except costa, lower margin sinuous, accompanied with narrow, dark brown line; dorsal bar white, at basal 1/3 of dorsum, dentiform, accompanied with dark brown bar along upper margin; marginal area dark brown; elongate scales of fringe pale reddish brown, with dark brown tips; hairy scales of fringe pale yellowish gray. Hindwing pale grayish orange; fringe yellowish gray.

*Abdomen*: Male tergum VIII sclerotized, subtrapezoidal, narrower caudally, emarginated posteriorly, with dense pores on posterior 1/3 and long process posterolaterally; male sternum VIII subrectangular, with short coremata posterolaterally.

*Male genitalia* (Figs 42–46): Tegumen round posteriorly, nearly parallel laterally, with an oval opening posteromedially; tuba analis as broad as vinculum. Valva divided into two portions; costal portion as long as tegumen, broad at basal 1/3, narrowed to cucullus, with a rectangular projection and a triangular projection at distal 2/5 of dorsal and ventral area respectively; cucullus digitate, with shallow bulge basally; saccular portion 1/2 as long as costal portion, obovate. Anellus extending to basal 5/8 of phallus. Vinculum elongate, subrectangular, as long as costal portion of valva, with T-shaped sclerotization medially; saccus 2/3 as long as vinculum, narrowed to apex. Phallus straight, broadened on basal 1/3.

Female genitalia not examined.

#### Type.

Holotype: female, “Holo-type” [round label with red borders], “Bartica, Brit[ish] Guiana. Parish. 2.13”, “*euryarga* Meyr.” [hand-written], “*Philonome euryarga* 1/1 Meyr[ick] E. Meyrick det. in Meyrick Coll.”, BMNH.

#### Material examined.

**French Guiana:** Régina: Nouragues Research Station (Lt: 4.1, Ln: 52): 1♂, 19 January 2010 (C. Lopez-Vaamonde), DNA Barcode LNOUA669-10, ID#: CLV76410, [GSN] USNM 34622, USNM.

#### Distribution.

Guyana and French Guiana.

### Philonome albivittata

Taxon classificationAnimaliaLepidopteraLyonetiidae

Sohn, Davis & Lopez-Vaamonde sp. n.

http://zoobank.org/EC0040A3-49DF-48BF-AB11-B03C618B363B

Figs 9 , 47–48

#### Diagnosis.

This species is similar to another congener, *Philonome euryarga* Meyrick in overall external appearance, but differs from the latter in having darker hindwings. Their male genitalia possess several distinct differences including the tegumen with lateral projections in *Philonome albivittata*; the saccus present only in *Philonome euryarga*; and in the form of the costal portion of valva (Figs 41 *vs.* 46).

#### Adult

(Fig. 9). *Head*: Vertex orange, intermixed with pale orange scales anteriorly and posteriorly and with dark brown scales laterally; scales on dorsum of occiput dark brown, orange on basal 1/4; scales between antennal scapes lustrous pale orange. Frons silvery white, concave at center. Antenna 4/5 as long as forewing; scape as long as diameter of eye, orange dorsally, silvery white anterolaterally and ventrally, intermixed with grayish brown scales apically; flagellomeres pale orange dorsally, lustrous pale yellow ventrally; 1st and 2nd flagellomeres intermixed with grayish brown scales dorsoapically. Labial palpus 1/2 as long as antennal scape, lustrous pale yellow.

*Thorax*: Patagium lustrous pale yellow; tegula white, intermixed with pale orange scales basally; mesonotum white on anterior half, lustrous reddish brown on posterior half, with a dark brown transverse band medially. Foreleg with coxa lustrous pale yellow; femur lustrous dark grayish brown laterally, lustrous pale yellow mesally; tibia and tarsus dark brown dorsally, pale grayish yellow ventrally. Midleg with coxa lustrous pale yellow; femur lustrous pale orange dorsally, lustrous pale yellow laterally and ventrally; tibia pale orange, intermixed with dark brown scales dorsally; tarsomeres orange dorsally, pale orange ventrally. Hindleg with coxa and femur lustrous pale orange; tibia pale brownish orange dorsally, pale orange ventrally, with stiff piliform scales; tarsomeres pale orange. Forewing length 2.8–3.1 mm (n = 3); reddish brown; costa brown; longitudinal fascia white, spanning entire costal area except costa; lower margin sinuous, accompanied with narrow, dark brown line; dorsal bar white, at basal 1/3 of dorsum, dentiform, accompanied with dark brown bar along upper margin; marginal area dark brown; fringe brownish gray. Hindwing and fringe brownish gray.

*Abdomen*: Male tergum VIII rectangular; male sternum VIII subrectangular, with oblique furrow and short coremata laterally.

**Figures 47–56.**
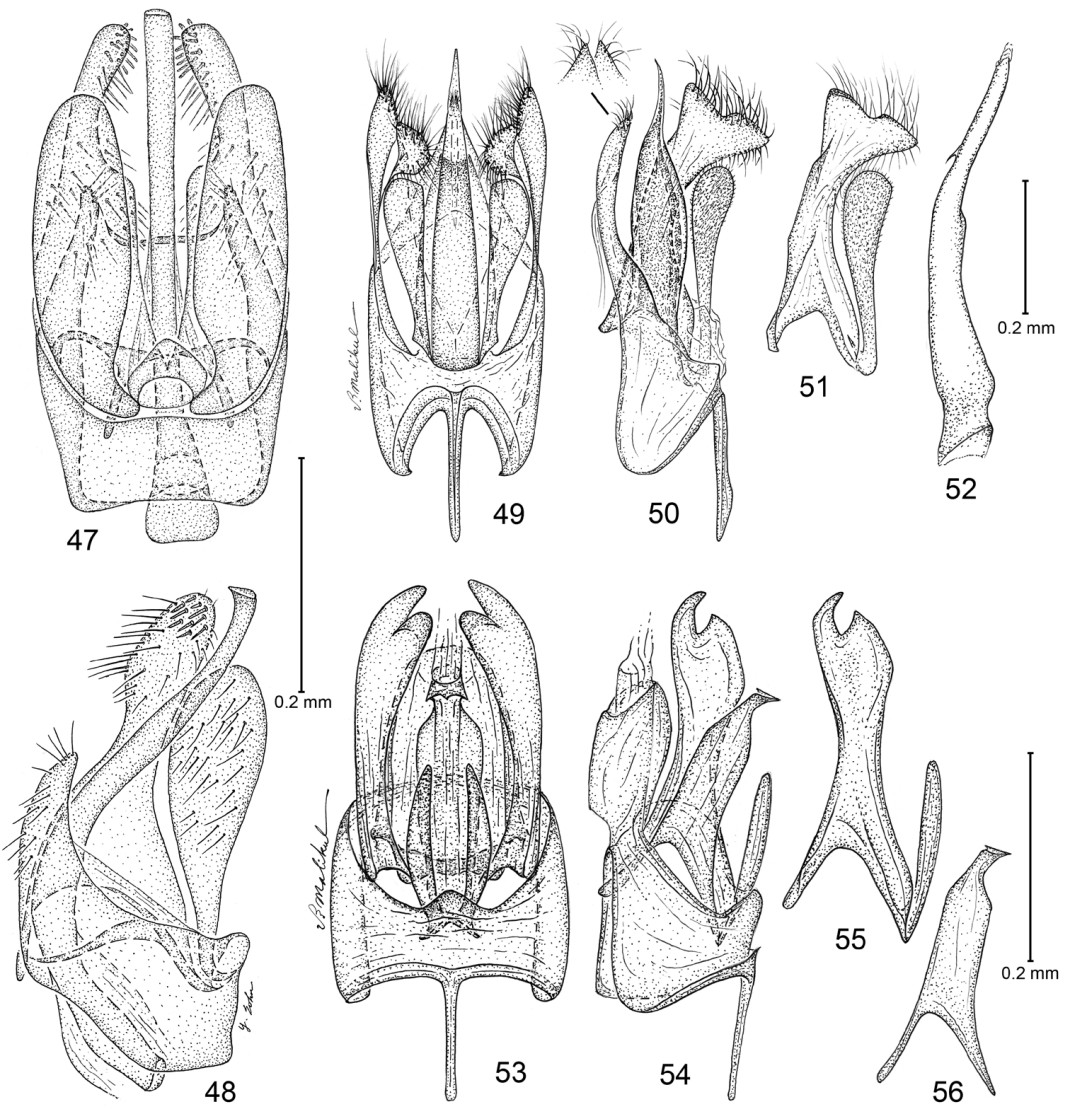
*Philonome*, male genitalia. **47**–**48** *Philonome albivittata* **47** Ventral view of genital capsule and phallus **48** Lateral view **49**–**52** *Philonome penerivifera* **49** Ventral view of genital capsule and phallus **50** Lateral view, with ventral detail of uncus **51** Lateral view of valva **52** Phallus **53–56** *Philonome rivifera*. **53** Ventral view of genital capsule and phallus **54** Lateral view **55** Lateral view of valva **56** Phallus.

*Male genitalia* (Figs 47–48): Tegumen subtrapezoidal, with sparsely setose, digitate projection posterolaterally. Valva divided into two portions; costal portion 2× longer than tegumen, broad basally, narrowed to cucullus; cucullus spatulate, narrowly round apically, sparsely setose, with short, spiniform setae in apical 1/4; saccular portion 1.5× as long as tegumen, elongate, obovate, sparsely setose. Anellus funnel-shaped, broadened basally. Juxta with an ovate bulge and a ridge connected to anellus. Vinculum rectangular, slightly concave anteriorly. Phallus slender and of even diameter on posterior 4/5, enlarged subtriangularly around ductus ejaculatorius.

Female unknown.

#### Types.

Holotype: male, “ID#: CLV10410 [red letters] French Guiana: [Régina, Nouragues Research Station] [Lt:4.1 Ln:52/] Carlos Lopez Vaamonde 16-Jan-2010 DNA Barcode LNOUA009 [sic: 946] -10 [green letters in blue row]”, “Genitalia slide DRD ♂ USNM 34623” [green label], USNM. Paratype: **French Guiana:** Régina: Nouragues Research Station (Lt:4.1, Ln:52): 1♂, 20 January 2010 (C Lopez-Vaamonde), “ID#: CLV94410”, “DNA Barcode LNOUA849-10”, [GSN] 34625, USNM.

#### Distribution.

French Guiana.

#### Etymology.

The species name is derived from the Latin adjectives, ‘albus’ and ‘vittatus’, meaning “white” and “banded” respectively, and refers to the white longitudinal band on the forewing of this new species.

### Philonome penerivifera

Taxon classificationAnimaliaLepidopteraLyonetiidae

Sohn & Davis sp. n.

http://zoobank.org/48A07D44-E6AC-4D0B-AD2F-17863CB24E88

Figs 10 , 49–52 , 61–62

#### Diagnosis.

This species is indistinguishable from *Philonome rivifera* in external appearance. Both species can be clearly distinguished from each other by the male genitalia (Table 2), including distal margin of cucullus shallowly concave in *Philonome penerivifera* but deeply emarginated in *Philonome rivifera*; tegumen triangular in *Philonome penerivifera* but subrectangular in *Philonome rivifera*; and lateral area of vinculum less protruding in *Philonome penerivifera* than in *Philonome rivifera*.

#### Adult

(Fig. 10). *Head*: Vertex brownish white or pale orange on posterior 2/3, pale yellowish white on anterior 1/3; scales on interspace between antennal scapes yellowish white; frons lustrous yellowish white; scales on occiput pale orange, with dark brown tips dorsally, pale yellowish white laterally. Antenna 3/5 as long as forewing; scape pale grayish orange dorsally, lustrous yellowish white ventrally; flagellomeres pale orange dorsally, yellowish white ventrally. Labial palpus 1/2 as long as maxillary palpus, dark grayish brown laterally, lustrous yellowish white mesally. Maxillary palpus yellowish white.

*Thorax*: Scales of patagium pale orange, with dark brown tips; tegula reddish brown basally, paler to apex, pale orange apically; mesonotum pale orange, transversely intermixed with dark brown scales at middle. Fore- and midlegs with coxa lustrous yellowish white; femur, tibia, and tarsomeres dark brown dorsally, lustrous yellowish white laterally and ventrally. Hindleg pale brownish gray dorsally, lustrous yellowish white laterally and ventrally. Forewing length 3.2–3.6 mm (n = 2), coloration and patterns similar to *Philonome rivifera*. Hindwing dark brownish gray; fringe brownish gray on costal and apical area, yellowish gray along posterior margin.

*Abdomen*: Terga pale grayish orange or pale grayish brown; sterna lustrous, white or pale orange.

*Male genitalia* (Figs 49–52): Tegumen triangular, with bifid, setose apex; tuba analis arising from dorsoposterior area of tegumen. Valva divided into two portions; costal portion broadened in basal 1/2, triangular in distal 1/3; distal margin of cucullus slightly emarginated medially, with dense long setae; saccular portion elongate, spatulate, densely setose. Anellus conical, nearly as long as phallus, with minute thorns on interior wall. Vinculum elongate-subrectangular, with semi-elliptical emargination anteromedially; saccus elongate, as long as uncus. Phallus slightly curved at distal 1/3, broadened anteriorly.

*Female genitalia* (Figs 62–62): Apophyses posteriores 2.5× longer than apophyses anteriores. Lamella antevaginalis conical, obliquely truncate apically, setose posterolaterally. Ductus bursae as long as corpus bursae, narrow; inception of ductus seminalis at posterior 1/4 of ductus bursae; ductus seminalis coiled. Corpus bursae obovate, with scattered microscopic thorns.

**Figures 57–60.**
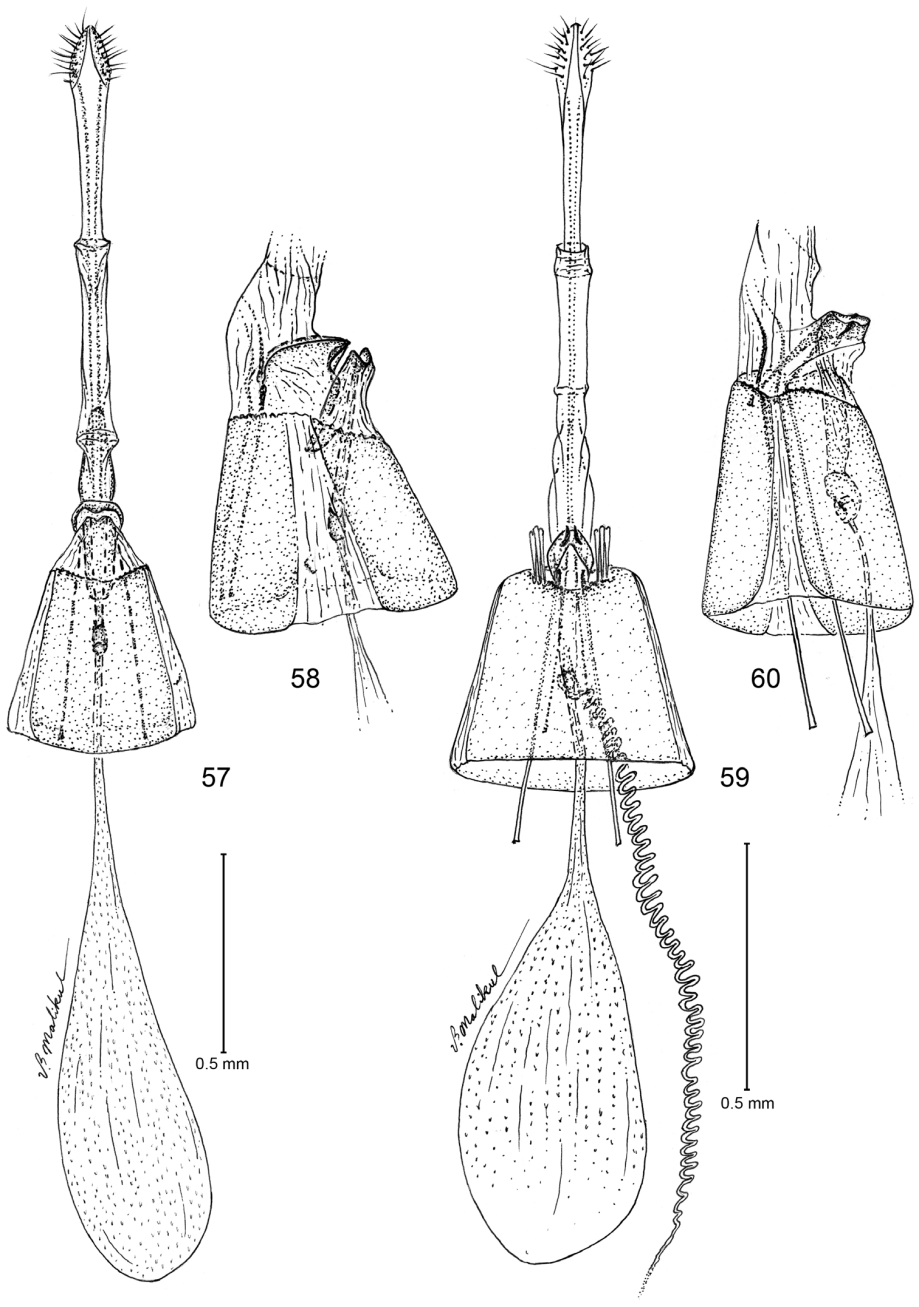
*Philonome*, female genitalia. **57**–**58** *Philonome nigrescens* **57** Ventral view **58** Lateral view of segment 8 and sterigma **59**–**60** *Philonome clemensella* **59** Ventral view **60** Lateral view of segment 8 and sterigma.

**Figures 61–66.**
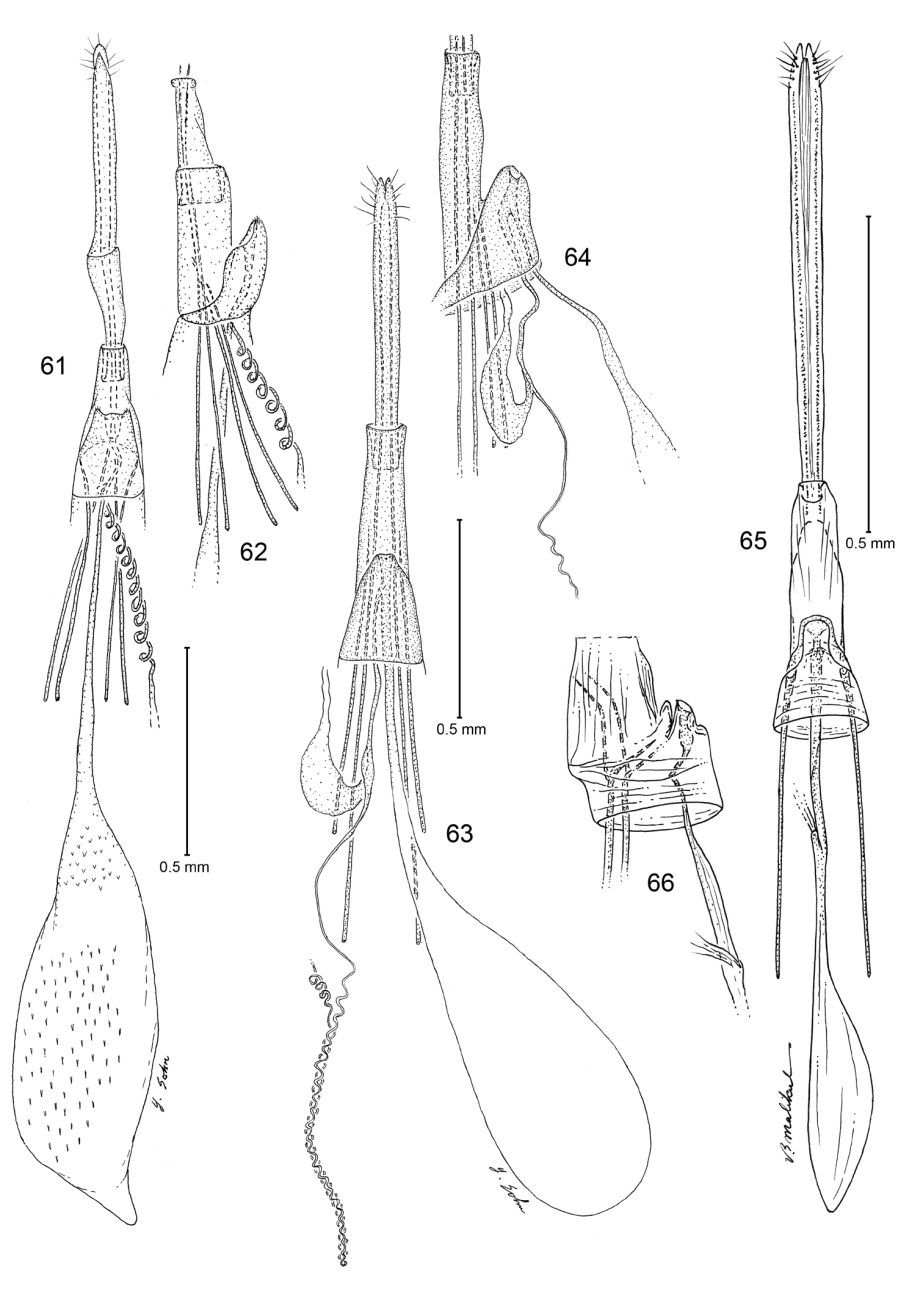
*Philonome*, female genitalia. **61**–**62** *Philonome penerivifera* **61** Ventral view **62** Lateral view of segment 8 and sterigma **63**–**64** *Philonome kawakitai* **63** Ventral view **64** Lateral view **65**–**66** *Philonome rivifera* **65** Ventral view **66** Lateral view of segment 8 and sterigma.

#### Types.

Holotype: male, “Holo-Type” [circular label with red border], “Para Brazil Parish 6 -19.”, “*Philonome rivifera* 7/17 Meyr. E. Meyrick det. in Meyrick Coll.” [old label attached before this study], “Meyrick Coll. B.M. 1988-290.”, “B.M. ♂ Genitalia slide No. 32828”. Paratypes (1♂, 4♀, 1 ex.): **Brazil:** Amazonas: Munaos [= Manaus], 2♀, “11.19” (Parish), BMNH. Tefé, 1 ex., “1.20” (Parish), BMNH. Federal District: Planaltina (15°35’S, 47°42’W, alt. 1000m): 1♀, 3 May 1984 (VO Becker), “BECKER 56394”, VOB; 1♀, 15 June 1985, “BECKER 57636”, [GSN] USNM 34615, USNM. Pará: Óbidos, 1♂, “9.19” (Parish), BMNH.

#### Distribution.

Brazil (Amazonas, Federal District, Pará).

#### Etymology.

The species name is derived from the Latin prefix ‘pene (= paene)’, meaning “almost”, and the preexisting species name, *rivifera*, and refers to the overall similarity of this species to *Philonome rivifera*.

#### Remarks.

The holotype and three paratypes of *Philonome penerivifera* in the BMNH collection were misidentified as *Philonome rivifera* by Edward Meyrick.

### Philonome kawakitai

Taxon classificationAnimaliaLepidopteraLyonetiidae

Sohn, Davis & Lopez-Vaamonde sp. n.

http://zoobank.org/463A273D-CFFF-418E-8325-47B034B42A19

Figs 11 , 63 , 64

#### Diagnosis.

The female genitalia of *Philonome kawakitai* is similar to those of *Philonome penerivifera* but differ from the latter in having the lamella antevaginalis of the seventh sternite more rounded (obliquely truncate posteriorly in *Philonome penerivifera*) and in the absence of microscopic spicules in the corpus bursae. *Philonome kawakitai* is distinguished from *Philonome curvilineata*, *Philonome penerivifera*, and *Philonome rivifera* in having the dorsal bar not reaching the dorsal margin and the complete subterminal line on the forewing.

#### Adult

(Fig. 11). *Head*: Vertex pale brown, sparsely intermixed with dark brown scales posterolaterally, pale orange on anterior 1/3; frons lustrous pale grayish yellow. Antenna 8/9 as long as forewing; scape pale orange dorsally, lustrous yellowish white ventrally; first flagellomere dark brown dorsally, pale yellow ventrally; second to 9^th^ flagellomeres pale orange dorsally, pale yellow ventrally; remaining flagellomeres pale grayish yellow. Labial palpus 1/2 as long as maxillary palpus, dark yellowish brown. Maxillary palpus dark yellowish brown.

*Thorax*: Scales of patagium pale orange, with dark brown tips; tegula pale brown on basal 1/3, pale orange on distal 2/3; mesonotum pale orange, sparsely intermixed with dark brown-tipped scales. Fore- and midlegs with coxa lustrous pale orange; femur, tibia, and tarsomeres dark brown mesally, lustrous yellowish white laterally. Hindleg with coxa pale orange; femur and tibia pale yellowish grayish dorsally, pale yellow ventrally; tarsomeres pale grayish yellow. Forewing length 3.8 mm (n = 2), reddish brown, paler along dorsal area; costal area pale orange, intermixed with dark brown scales densely on basal 1/3 and sparsely on distal 1/3; longitudinal fascia continuous to near termen, convex and narrowed at distal 1/3, white, juxtaposed with slender black line along lower border; dorsal bar as triangular patch on anterior half, combined to longitudinal fascia and as slender, intermittent, black line on posterior half; subterminal line connecting distal 1/8 of costa and tornus, dark brown, intermittent; fringe pale brownish gray, with three dark brown, transverse lines. Hindwing dark brownish gray; fringe pale brownish gray.

Male unknown.

*Female genitalia* (Figs 63–64): Apophyses posteriores 2.2× longer than apophyses anteriores. Lamella antevaginalis conical and narrowly rounded caudally. Ductus bursae as long as corpus bursae, narrow; inception of ductus seminalis at posterior 1/8 of ductus bursae; ductus seminalis coiled in distal portion. Corpus bursae obovate, without signum or microscopic spicules.

#### Type.

Holotype: female, “FRENCH GUIANA: Nouragues Nature Reserve Nouragues Research Station Sep[tember]-07-2010 collected by light trapping”, “Genitalia slide DRD ♀ USNM 34652” [green label], USNM.

#### Distribution.

French Guiana.

#### Etymology.

The species name is a patronym in honor of Dr. Atsushi Kawakita who collected the holotype.

### Philonome rivifera

Taxon classificationAnimaliaLepidopteraLyonetiidae

Meyrick, 1915

Figs 12–13 , 53–56 , 65–66

Philonome rivifera Meyrick, 1915: 251.

#### Adult

(Figs 12–13). *Head*: Vertex orange; frons lustrous pale orange, concave at center; scales on dorsum of occiput pale orange, dark purplish brown on apical 1/4; scales between antennal scapes, elongate, pale orange. Antenna 2/3 as long as forewing; scape as long as diameter of eye, lustrous pale orange, paler ventrad, narrowly suffused with orange dorsally; flagellomere I–VII pale reddish brown dorsally, lustrous pale orange ventrally; the remaining flagellomeres lustrous pale orange dorsally, paler ventrad. Labial palpus 3/4 as long as maxillary palpus, silvery white on interior surface, lustrous pale yellow on exterior surface, suffused with pale grayish orange apically. Maxillary palpus pale grayish brown.

*Thorax*: Patagium pale orange, tinged with dark brown distally; tegula lustrous pale yellow, intermixed with brown-tipped, orange scales basally; mesonotum pale orange, anterior 1/3 and posterior 1/3 lustrous pale yellow, with a narrow transverse band of dark brown-tipped scales. Fore- and midlegs with coxa lustrous pale yellow; femur pale orange dorsally, lustrous pale yellow laterally and ventrally; tibia and tarsomeres pale brown dorsally, pale orange ventrally. Hindleg with coxa and femur lustrous pale orange; tibia lustrous pale orange, with long piliform scales ventrally; tarsomeres orange dorsally, pale orange ventrally. Forewing length 2.8–4.6 mm (n = 3), reddish brown in medial area, orange in terminal 1/3 of costal area, pale orange in basal 2/3 of costal area and in basal 1/2 of dorsal area; longitudinal fascia white, continuing to subapical area, accompanied with a slender, dark brown line along lower margin, curved to costa at terminal 1/3; dorsal bar white, narrow, connected to white spreading on dorsum; distal area of costa, termen, and apical area densely irrorated with dark brown; elongate scales of fringe pale grayish brown, with dark brown tip; piliform scales of fringe pale orange. Hindwing dark grayish brown; fringe purplish gray.

*Abdomen*: Terga grayish brown; sterna pale orange. Male tergum VIII rectangular; male sternum VIII subrectangular, broadly emarginated posteriorly; coremata absent.

*Male genitalia* (Figs 53–56): Tegumen 5/7 as long as valva, elliptical, convex anterolaterally, with round opening apically; tuba analis arising from apical opening. Valva divided into two portions; costal portion broadened in basal 1/3 and distal 1/3; cucullus divided into two projections apically, one falcate and the other small, triangulate; saccular portion narrow, digitate. Juxta trapezoidal. Vinculum wide, rectangular, convex posteromedially, with small protrusion laterally; saccus slender, as long as saccular portion of valva. Phallus of even width on basal 4/5, narrowed on distal 1/5, diverging into two projections basally.

*Female genitalia* (Figs 65–66): Papillae anales narrow, semi-elliptical; apophyses posteriores 1.2× longer than apophyses anteriores. Lamella postvaginalis quadrate. Lamella antevaginalis cylindrical. Ductus bursae narrow; inception of ductus seminalis present at middle of ductus bursae. Corpus bursae narrow, elliptical.

#### Types.

Lectotype (designated here): male, “LECTO-TYPE” (round label with indigo boarders), “Bartica Brit[ish] Guiana Parish. 2.13”, “Meyrick Coll. B.M. 1938-290.”, “*Philonome rivifera* 10/17 Meyr[ick] E. Meyrick det. in Meyrick Coll.”, BMNH. Paralectotypes: **Guyana:** same data as lectotype: 1♂, 4♀, [GSN] BM 31892 (♂) & BM 32829 (♀), BMNH.

#### Distribution.

Guyana.

#### Remarks.

Meyrick (1915) described *Philonome rivifera*, based on eight specimens. Only six of those syntypes have been located in the BMNH. The specimen labels indicate that one of those was selected as the lectotype. This designation, however, has never been published, and the same specimen is designated here as the lectotype of *Philonome rivifera*.

### Philonome spectata

Taxon classificationAnimaliaLepidopteraLyonetiidae

Meyrick, 1920

Fig. 15

Philonome spectata Meyrick, 1920: 359; Davis 1984: 25.

#### Adult

(Fig. 15). *Head*: Vertex reddish brown; frons pale orange. Antenna 3/4 as long as forewing; scape white, suffused with pale orange anterobasally; first five flagellomeres white; remaining flagellomeres pale grayish brown. Labial palpus and maxilary palpus pale orange.

*Thorax*: Patagium and mesonotum reddish brown; tegula white. Legs pale orange. Forewing length 2.3 mm (n = 1), reddish brown; longitudinal fascia white, covering most costal area, lower margin sinuous, accompanied with very narrow dark brown line; costa suffused with pale orange subbasally and in terminal 1/3; elongate scales of fringe around apex reddish brown with dark brown tips; piliform scales of fringe on terminal 1/4 of costa and on tornal area yellowish brown with dark brown tips. Hindwing lustrous, yellowish gray, paler to base; fringe pale yellowish gray.

*Abdomen*: Terga and sterna lustrous white.

Female genitalia not examined.

#### Type.

Holotype: female, “Holo-type” [round label with red borders], “Para Brazil Parish 7-19.”, “Meyrick Coll. B.M. 1938-290.”, “*Philonome spectata* 1/1 Meyr[ick] E. Meyrick det. in Meyrick Coll.”, BMNH.

#### Distribution.

Brazil (Pará).

#### Remarks.

Only the holotype of *Philonome spectata* is known to exist. It was not possible to examine this specimen and to illustrate the genitalia. This species can be distinguished from other congeners in lacking the dorsal bar on the forewing.

### Philonome sp.

Taxon classificationAnimaliaLepidopteraLyonetiidae

Fig. 14

#### Note.

Forewing length 4.1 mm (n = 1). This species is indistinguishable from *Philonome rivifera* in superficial appearance. Our DNA-barcoding data show that it is distinct from other congeners from French Guiana and *Philonome clemensella*, and may be genetically closest to *Philonome kawakitai* (Fig. 71). The only specimen of this species has its abdomen missing. Its description is pending until additional specimens are found.

**Figures 67–70.**
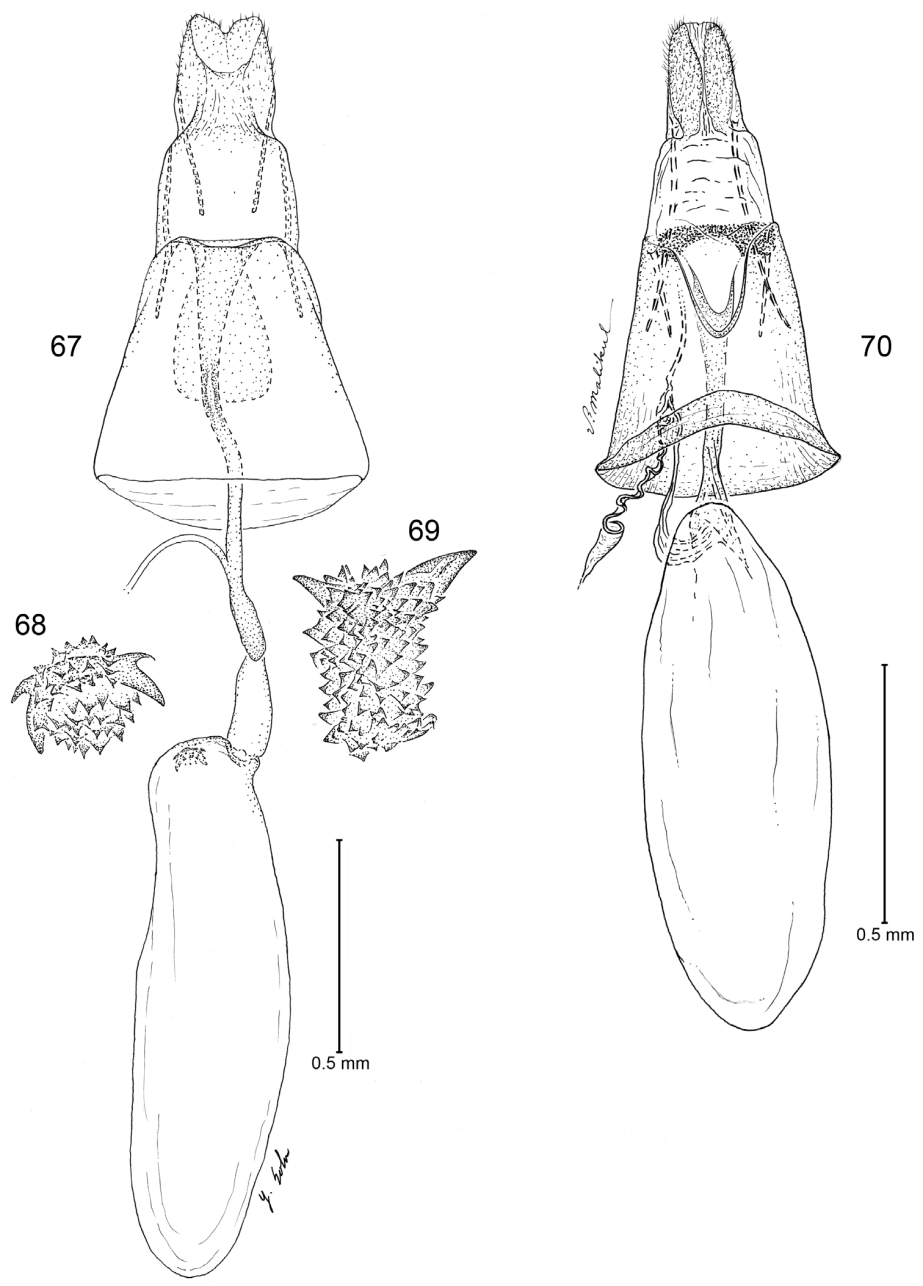
Female genitalia. **67**–**69** *Argyresthia luteella*. **67** Ventral view **68** Enlarged view of signum, ventral view **69** anterior view of Fig. 64 **70** *Elachista albella*, ventral view.

**Figure 71.**
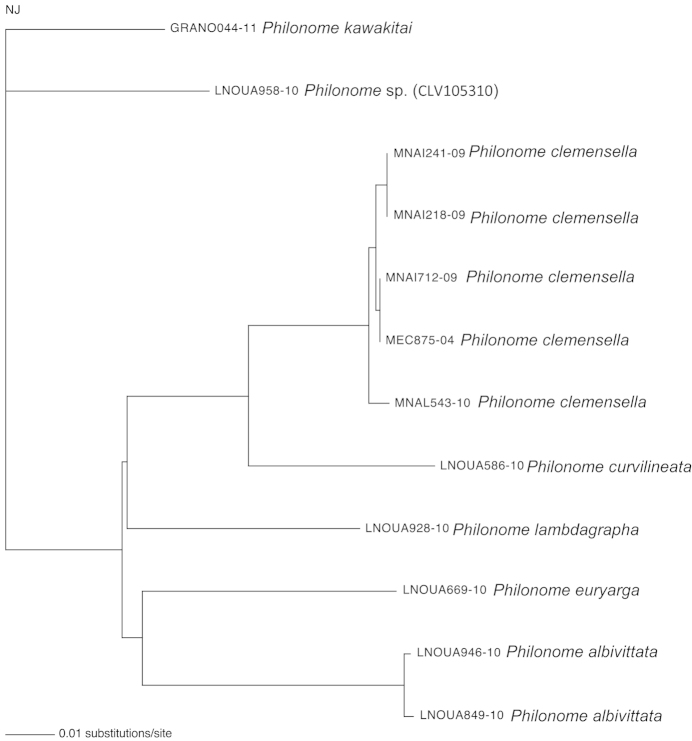
A Neighbor-Joining tree, generated under the K2P nucleotide substitution model, for the species of *Philonome*. Branch lengths represent the number of substitutions per site.

#### Material examined.

**French Guiana:** Régina: Nouragues Research Station (Lt: 4.1, Ln: 52): 1♂, 24 January 2010 (C. Lopez-Vaamonde), DNA Barcode LNOUA958-10, ID#: CLV105310, USNM.

#### Distribution.

French Guiana.

### Revised status of *Philonome luteella* (Chambers)

*Philonome luteella* (Chambers) was originally the type species of *Eurynome* Chambers, 1875. The generic name was found to be preoccupied by *Eurynome* Leach, [1814] and was replaced with *Busckia* Dyar, 1903. McDunnough (1939) synonymized *Busckia* with *Philonome*. Our examination revealed that this species is actually a member of *Argyresthia* Hübner, [1825] (Argyresthiidae). Therefore, *Eurynome* Chambers, 1875 and its replacement name, *Busckia* Dyar, 1903, are here synonymized with *Argyresthia* Hübner, [1825].

### Argyresthia

Taxon classificationAnimaliaLepidopteraYponomeutidae

Hübner, [1825]: 422.

#### Type species.

*Phalaena goedartella* Linnaeus, 1758, by subsequent designation by Busck (1907).

*Eurynome* Chambers, 1875: 304. A junior homonym of *Eurynome* Leach, [1814] [Crustacea]. **syn. n.** Type species: *Eurynome luteella* Chambers, 1875, by monotypy.

*Busckia* Dyar, 1903: 563. An objective replacement name of *Eurynome* Chambers, 1875. **syn. n.**

### Argyresthia luteella

Taxon classificationAnimaliaLepidopteraYponomeutidae

(Chambers, 1875) comb. n.

Figs 16 , 67–69

Eurynome luteella Chambers, 1875: 304.
Busckia luteella (Chambers): Dyar 1903: 563.
Philonome luteella (Chambers): McDunnough 1939: 100; Davis 1983: 8.

#### Adult

(Fig. 16). Head missing from the holotype. Chambers (1875) stated that “head, eye caps and palpi white, the latter stained with yellowish”.

*Thorax*: Patagium pale saffron yellow (Chambers 1875); mesonotum yellowish white, suffused with pale orange laterally and on posterior 1/3. Foreleg with coxa pale orange; other segments missing from holotype. Mid- and hindlegs with coxa, femur, and tibia pale orange dorsally, lustrous yellowish white ventrally; tarsomeres pale grayish brown dorsally, lustrous yellowish white ventrally. Forewing 3.4 mm (n = 1), lustrous yellowish white; basal and apical areas yellowish orange; antemedian, postmedian, and subterminal fasciae yellowish orange, oblique, indistinctly outlined; fringe yellowish orange on basal 1/3, purplish gray on distal 2/3. Hindwing lustrous yellowish white; fringe yellowish gray.

Male unknown.

*Female genitalia* (Figs 67–69): Papillae anales subrectangular, slightly protruding dorsolaterally. Apophyses posteriores nearly as long as apophyses anteriores including basal fork. Ostium bursae on posterior margin of sternite VIII. Ductus bursae as long as corpus bursae, funnel-shaped on posterior 2/5; antrum extending caudally over 1/3 of ductus bursae, cylindrical. Corpus bursae elongate, elliptical; signum at anterior area of corpus bursae, denticulate, with two diverging, large, spiniform sclerites posteriorly.

#### Type.

Holotype: female, “Kentucky [sic] Chambers”, “Type 14964” [red label], “*Eurynome luteella* Chambers” [hand-written on folded paper], “Genitalia slide MCZ-L122 Prep. by JC Sohn” [label with black border lines].

#### Distribution.

Western United States (Colorado). Chambers (1875) stated “Spanish Bar”, now Fall River in Larimer County, Colorado, as the collecting locality. On the label of the holotype of *Philonome luteella*, “Kentucky” was given as collecting locality with strikethrough mark indicating that the locality is not correct.

#### Remarks.

The forewing pattern and the female genital morphology of *Eurynome luteella* suggest that it is not congeneric with *Philonome*. Its forewing pattern is similar to some species of *Argyresthia*, especially *Argyresthia cupressella* Walsingham, 1891, and *Argyresthia freyella* Walsingham, 1891. The female genitalia of *Eurynome luteella* include a denticulate signum of which the shape is typical for *Argyresthia*. This species, consequently, has been reassigned to *Argyresthia*.

## Discussion

### Systematic position

*Philonome* has been associated frequently with *Bucculatrix*, since Chambers (1875). Both genera were placed in Lyonetiidae (Meyrick 1915, 1920; Barnes and McDunnough 1917; Forbes 1923). *Philonome* was retained within Lyonetiidae in recent checklists (e.g. Davis 1983; Poole and Gentili 1996), while *Bucculatrix* now constitutes its own family, Bucculatricidae (Davis and Robinson 1998). Heppner (1984, 2011) assigned *Philonome* to Bedelliinae (now Bedelliidae) without explanation. Recently, Sohn et al. (2013) included *Philonome clemensella* as an outgroup in their phylogenetic analyses for Yponomeutoidea and they proposed that the genus belongs to Tineoidea (Fig. 1). In their resulting tree, *Philonome* was nested strongly within a monophyletic Tineidae *sensu* Regier et al. (2015). Several interrelationships of the genera included in the clade were unresolved from the study, but *Philonome* was further nested within a subclade of Tineidae (Fig. 1: A) that also included *Tinea* Linnaeus, 1758, *Perissomastix* Warren & Rothschild, 1905, *Nemapogon* Schrank, 1802, *Euprora* Busck, 1906, *Erechthias* Meyrick, 1880, *Scardiella* Robinson, 1986, *Harmaclona* Busck, 1914, and *Opogona* Zeller, 1853. The interrelationships of these genera were largely unresolved. In the best Maximum Likelihood tree constructed by Sohn et al. (2013), *Philonome* was grouped with a pair, *Tinea columbariella* Wocke, 1877 and *Perissomastix* sp., but this grouping was very weakly supported. Consequently, Sohn et al. (2013) identified *Philonome clemensella* as an unstable or rogue taxon.

Despite the strong support from molecular data, the tineid association of *Philonome* has never been addressed with morphological studies. Among the morphological characters associating *Philonome* with Tineidae are the reduced, naked haustellum with unassociated galeae, 5-segmented maxillary palpi, and vein Rs_4_ terminating on costa before the forewing apex. It now appears that *Philonome* is most allied to the tineid subfamily Hieroxestinae also on the basis of morphological similarities. These include the wedge-shaped head (lateral view), vestiture of head partially consisting of appressed, laminate scales, and elongate scape without pecten. Previous association of this genus with *Bucculatrix* and Lyonetiidae was most likely decided largely by the presence of the broadly scaled antennal scape which forms an eyecap, a feature absent or poorly developed in Tineidae but typical for the latter two families. Eleven genera and 289 species are now recognized globally within Hieroxestinae, with 180 species assigned to *Opogona* (Robinson 2009). Within this subfamily, *Philonome* appears most similar morphologically to *Oinophila* Stephens, 1848, a holarctic genus currently restricted to two species. In particular, the head vestiture of both genera share unusual specializations not observed in other Hieroxestinae. The adult heads of Hieroxestinae typically possess a smooth, broad scaled frons and occiput, and a rough vertex consisting of a tuft of erect, piliform scales. The heads of *Philonome* and *Oinophila* are unusual in having the piliform scales of the vertex divided by a narrow, transverse band of broad, flat scales extending between the bases of the antennae (Davis 1978; Robinson and Tuck 1997). *Philonome* and *Oinophila* also possess similar wing venation, with the R vein lacking in the forewing and Rs with 4 branches. The heads of both genera possess a relatively raised vertex, and the rudimentary mandibles are better developed than in other genera of the subfamily. The antennal scape of *Oinophila* differs from that of *Philonome* in being more slender, smoothly scaled, and not formed into an eyecap. The female genitalia of *Philonome* differ from other known Hieroxestinae by lacking a signum in the corpus bursae.

Despite some possible synapomorphies between *Philonome* and the Hieroxestinae, we find them insufficient for a final taxonomic placement, and therefore leave the genus unplaced in Tineidae.

### DNA barcoding

Figure 71 shows a neighbor joining tree based on the DNA barcode sequences for 12 individuals of *Philonome* available at BOLD systems (www.barcodinglife.org). The resulting tree and the distance matrix (Table 3) indicate the presence of seven unique taxonomic units which can be assigned to the separate Barcode Index Numbers (BINs: Ratnasingham and Hebert 2013). These include two previously known species of *Philonome*; *Philonome clemensella* (five individuals) and *Philonome euryarga* (one individual); four species described in this paper, *Philonome albivittata*, *Philonome curvilineata*, *Philonome kawakitai*, and *Philonome lambdagrapha* (all except *Philonome albivittata* based on singleton); and one species (Fig. 14: CLV105310) from French Guiana which cannot be named due to the loss of the abdomen.

**Table 3.** Kimura 2-parameter (K2P) distances (%) for barcode DNA sequences of the seven analyzed species in genus *Philonome*. Minimal pairwise distances between species are given for each species pair. Values in square brackets represent maximal intraspecific distances.

	*clemensella*	*curvilineata*	*euryarga*	*albivittata*	*lambdagrapha*	sp.	*kawakitai*
*clemensella*	[0.9]						
*curvilineata*	6.9						
*euryarga*	11.5	13.3					
*albivittata*	12	13.2	11.7	[0.3]			
*lambdagrapha*	10.7	12.4	11.7	11.2			
sp.	13	14.1	13.9	14.1	12.1		
*kawakitai*	12.2	12.4	11.4	12.8	12.1	7.9	

DNA barcodes of the seven species analysed are very distinctive (Fig. 71, Table 1). Indeed, DNA barcodes show high levels of interspecific genetic distance (Table 3). All species analysed show distinct DNA barcodes with a minimum interspecific pairwise genetic distance of 6.9% among all species. The maximum intraspecific genetic variation ranged from 0.9 to 0.3, much lower than interspecific distances, suggesting the existence of a barcode gap although current intraspecific sampling is too limited.

## Supplementary Material

XML Treatment for Philonome

XML Treatment for Philonome cuprescens

XML Treatment for Philonome wielgusi

XML Treatment for Philonome nigrescens

XML Treatment for Philonome clemensella

XML Treatment for Philonome lambdagrapha

XML Treatment for Philonome curvilineata

XML Treatment for Philonome euryarga

XML Treatment for Philonome albivittata

XML Treatment for Philonome penerivifera

XML Treatment for Philonome kawakitai

XML Treatment for Philonome rivifera

XML Treatment for Philonome spectata

XML Treatment for Philonome sp.

XML Treatment for Argyresthia

XML Treatment for Argyresthia luteella
